# Photoactivated Signaling Networks using DNA‐Based Synthetic Organelles as Biomimetic Protocells

**DOI:** 10.1002/anie.4889049

**Published:** 2026-05-01

**Authors:** Huiying Xue, Yunlong Qin, Yichen Han, Shijun Xu, Itamar Willner, Fan Xia, Fujian Huang

**Affiliations:** ^1^ State Key Laboratory of Geomicrobiology and Environmental Changes Faculty of Materials Science and Chemistry China University of Geosciences Wuhan China; ^2^ The Institute of Chemistry The Hebrew University of Jerusalem Jerusalem Israel

**Keywords:** DNA condensate, DNAzyme, liposome, photoresponsive, transcription

## Abstract

Membraneless organelles formed by phase‐separated nucleic acid or protein condensates play vital roles in regulating cellular functions. Integrating such synthetic organelles into protocell carriers remains a key challenge. Here, we introduce a method to assemble functional phase‐separated organelles within liposome protocells. Pre‐engineered nucleic acids are encapsulated with ligase in locked‐DNA‐nanopore modified protocells. Upon nanopore unlocking and Mg^2+^ influx, the nucleic acid constituents ligate into programmable polymer chains that crosslink into barcode‐modified condensates. Photoresponsive, caged nucleic acids hybridize with barcode tethers on two distinct organelles, forming a functional two‐organelle system in the protocells. Light‐induced uncaging releases an information‐transfer strand from one organelle, triggering intercommunication and reconfiguration of the partner organelle. By predesigning organelle compositions and transfer strands, the emergence of catalytic DNAzymes or transcriptional machinery in the organelle/protocell assemblies is demonstrated, resulting in dynamic structural reconfiguration of the organelles.

## Graphical abstract

Light‐triggered intercommunication between two types of organelle‐loaded liposomes lead to DNAzyme‐mediated on transcription‐guided structural reconfiguration of the organelles.

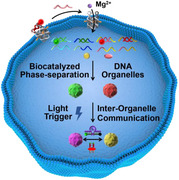

## 1 Introduction

Membraneless organelles consisting of intracellular phase‐separated nucleic acid or protein condensates play key roles in the spatiotemporal control of cell functionalities [1, 2, 3, 4]. While organelles are considered key constituents for the emergence of living matter [5], they provide concentrated compartmentalized nucleic acid, protein, and metabolite microenvironments regulating physiochemical cell functions, such as controlling protein or nucleic acid folding efficiencies [3, 6, 7, 8, 9, 10], providing distinct condensates for disposal of waste metabolites, and generation of orthogonally localized pH‐environments within the buffered cell containments. For example, organelles, such as nucleoli, Cajal bodies, and stress granules, were reported to regulate biogenetic transcription and post‐transcriptional modification [9, 11, 12, 13, 14, 15, 16, 17] and inter‐organelle signaling playing central roles in cellular transformations [18, 19]. Moreover, phase‐separated organelles were suggested to be involved in pathological events like fibrillization [13] or neurodegenerative diseases [20, 21, 22].

Substantial research efforts are directed towards the development of synthetic cells or protocells emulating native cell functions [23, 24, 25]. Diverse cell‐like containments, such as liposomes [26, 27, 28, 29, 30], polymersomes [31], dendrosomes [32], proteinosomes [33, 34, 35], water‐in‐oil droplets [36, 37], coacervate microdroplets [38, 39], or microcapsules [40, 41], were introduced as structural and functional reservoirs mimicking native cell frameworks. Particularly, efforts to compartmentalize synthetic cells and to engineer synthetic organelles [42], demonstrating programmed intercommunication between compartments [43] or organelles exhibiting native organelle‐like functionalities were reported. For example, light‐triggered growth of organelles in protocells was demonstrated by photochemical uncaging of a photoresponsive Y‐shaped DNA module and autonomous coacervation of the organelles in the water‐in‐oil droplets [44]. Also, annealing of programmable DNA constituents in water‐in‐oil droplets led to the guided formation of the self‐complementary Y‐shaped framework that underwent phase separation into condensates. In addition, a protein‐mediated photopolymerization of multi‐compartment coacervates mimicking organelle compartmentalization was reported [45]. By using two sets of programmable nucleic acid constituents, the formation of two kinds of organelles in the droplets, revealing light‐stimulated communication, was reported [46]. Moreover, substantial efforts were directed to the immobilization of enzymes in phase‐separated compartmentalized coacervates mimicking organelle functions in protocell model systems. These include the operation of an enzymatic cascade in confined coacervates assembled in microdroplet systems [47], the triggered programmable operation of enzymes in phase‐separated organelle like coacervates [48, 49], and the integration of enzyme‐loaded proteinosomes in coacervate protocells as functional organelle‐like constituents mediating biocatalytic morphological transitions of the protocells [50]. Also, the integration of photosynthetic organelles within liposomes for energy conversion, for example, ATP photosynthesis [51, 52], the cross‐interaction of enzymes embedded in organelles, the operation of enzyme cascades in confined protocell environments [53, 54], and the separation of transcription and translation machineries using gel‐based organelles [55] were demonstrated. In addition, transcription machinery‐loaded liposomes modified in their membrane boundaries with α‐hemolysin pores were employed as functional protocell mimicking the native cytoskeleton. Permeation of the NTPs through the membrane pores triggered the transcription machinery, synthesizing RNA origami tiles that self‐assemble into cytoskeleton‐like fibers [56]. Furthermore, phase‐separation of transcription machinery generated aptamer‐modified RNA condensates and the guided compartmentalization of protein‐aptamer complexes in the condensates provided biomimetic cell‐like functionalities [57]. Also, DNA‐mediated self‐organization of polymeric nanocomposites acted as artificial interconnected synthetic organelles [58]. Intriguing efforts include the synthesis of artificial organelles and their integration within native cells as constituents controlling cell functions [59]. These efforts demonstrate future potential use of organelles for sensing and therapeutic applications. Nevertheless, despite the advances in developing synthetic organelle nanostructures in protocell assemblies, the design of synthetic organelles of enhanced functional complexities emulating native organelles is desirable. This includes the design of synthetic organelles in protocell environments exhibiting built‐in programmability to operate signaled structural, biocatalytic, and transcriptional functions. These issues are addressed in the present study.

**FIGURE 1:**
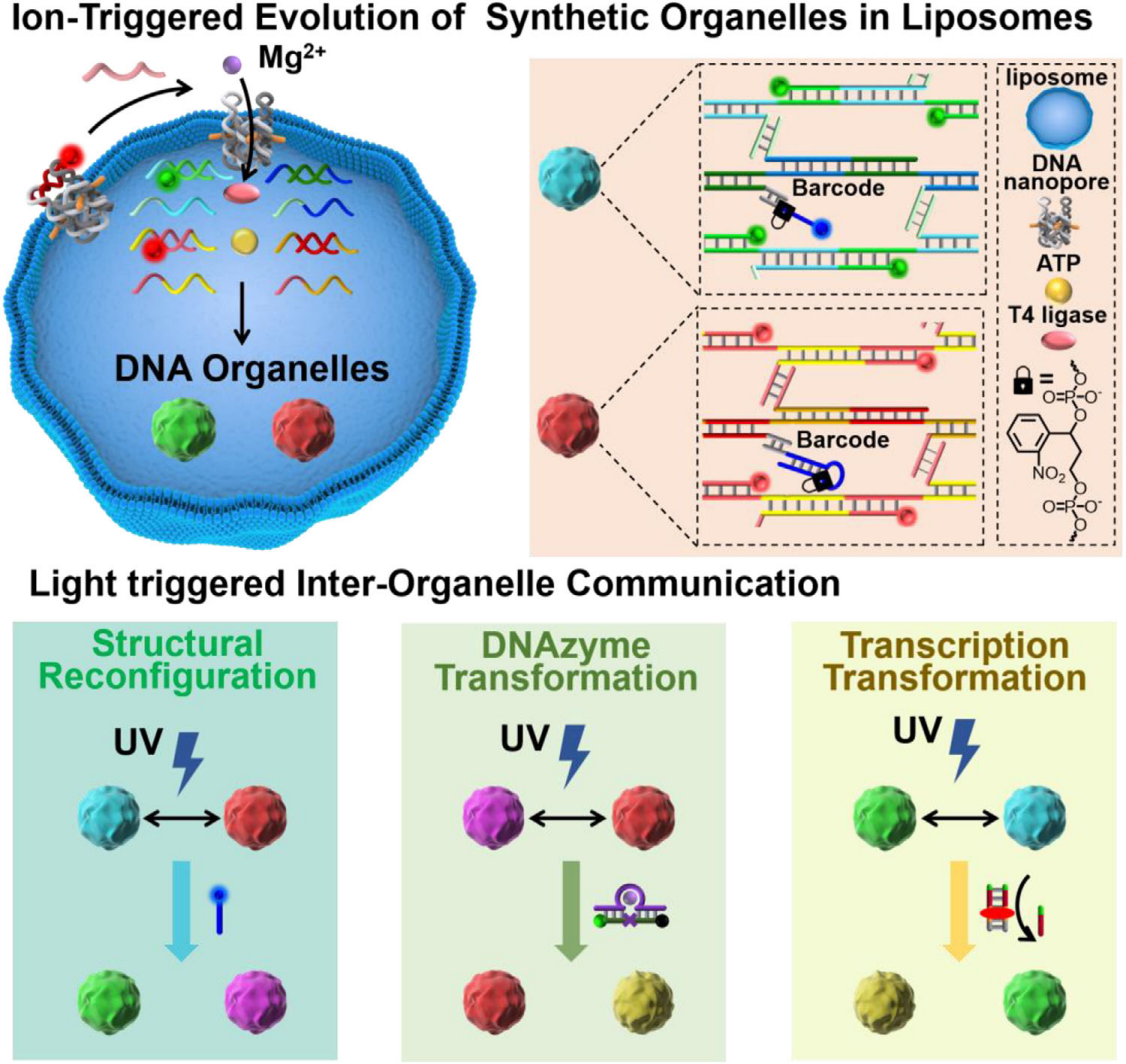
Schematic ion‐triggered formation of organelles in liposomes. Light‐triggered structural reconfiguration of the organelles, intercommunication between organelles and guided DNAzyme or transcriptional catalytic machineries in the protocell assemblies are demonstrated.

Here we wish to report on the nucleic acid‐based engineering of organelles in DNA origami nanopore‐functionalized liposome protocells, as schematically outlined in Figure 1. We make use of two previously reported concepts that described the ATP/ligase‐driven programmable phase separation of DNA constituents into condensates and the integration of origami nanopore channels in membranes as signal transfer units [60] to engineer a supermolecular liposome‐protocell assembly, enabling the triggered intra‐liposome dynamic phase separation of two intercommunicating functional organelles. A set of nucleic acid constituents and ligase are integrated in the liposome containment, while the DNA nanopores associated with the liposome membrane exist in a locked configuration. Unlocking of the nanopores facilitates the triggered transport of Mg^2+^‐ions into the liposome containment, the subsequent ligation of the nucleic acid constituents, and the guided phase separation of the resulting DNA chains into organelles, confined to the liposome protocells. The assembly of two types of barcode‐modified organelles in the liposome protocells is introduced. By appropriate functionalization of the organelle constituents with barcode tethers, light‐responsive sequence‐engineered nucleic acid structures are anchored to the barcodes through hybridization. Light‐induced uncaging of the nucleic acids frameworks, associated with the barcodes, leads to dynamic intercommunication between the two types of organelles, resulting in programmable structural reconfiguration and emerging catalytic functions within the organelles. These are reflected by the dynamic emergence of catalytic DNAzyme functions in the organelles, and the release of nucleic acid messenger strands activating transcription machineries, leading to the controlled structural reconfiguration of the organelles. The study introduces new dimensions towards the development of synthetic organelles in protocell systems, emulating organelle‐mediated signaling in living cells. The novel contributions of the study are reflected by the programmable engineering of functional organelles in liposome protocells. This enabled the light‐triggered intercommunication between the organelles leading to dynamic structural, catalytic, and transcriptional responses of the system emulating native cell functions.

## 2 Results and Discussion

**FIGURE 2:**
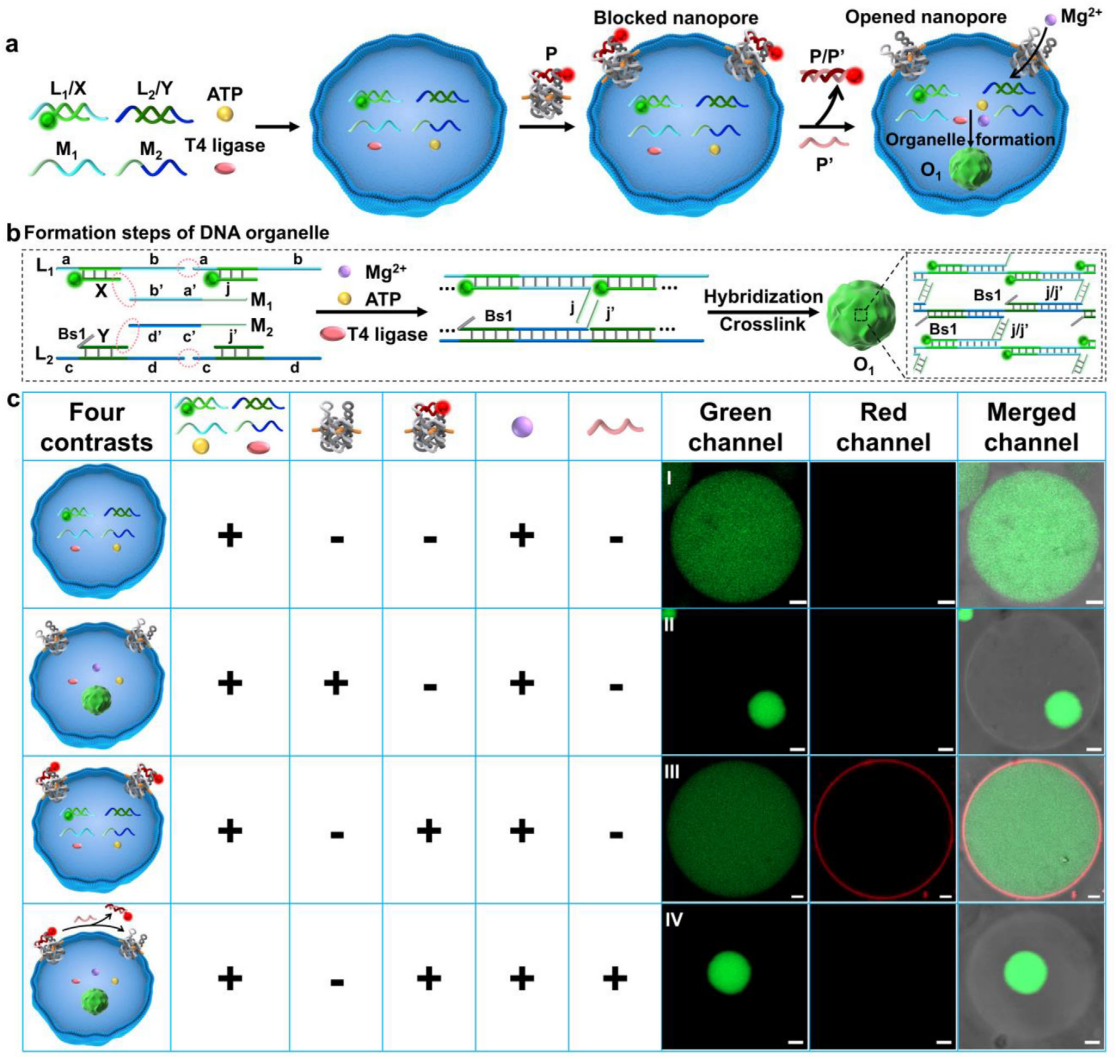
Assembly and characterization of a phase‐separated organelle in a liposome. (a) Integration of the set of constituents in the pore‐locked liposome assembly. (b) Detailed mechanistic display of the barcode tethered phase‐separated organelle in the liposome carrier, upon the fueled unlocking of the blocked nanopores associated with liposome boundaries and the transport of Mg^2+^‐ions activating the ligation of the liposome constituent yielding the barcode functionalized liposome organelles O_1_. (c) Confocal fluorescence microscopy images (scale bar = 2 µm) of the evolved organelle in the liposome carrier, and control systems.

Figure 2a,b depicts the method to assemble functional organelle‐like phase‐separated DNA condensates in giant unilamellar vesicle liposomes modified with DNA nanopore units. The liposomes were loaded with the DNA duplexes composed each of the L_1_ hybridized with the fluorophore (FAM)‐labeled X and L_2_ hybridized with Y, where strand L_1_ includes two toehold tethers a, b, and strand L_2_ includes two toehold tethers c, d. In addition, two single strands M_1_ and M_2_ were also included in the liposome containment, where M_1_ consists of two sub‐sequences a’ and b’ complementary to the toehold domain of L_1_ and is further functionalized with a free tether j. The strand M_2_ consists of two sub‐sequences c’, d’, complementary to the toehold tether associated with L_2_, and is further conjugated to the free tether, j’. T4 ligase and ATP were further included as a load in the liposome containment. The boundaries of the liposomes were modified with pre‐engineered DNA origami acting as nanopore units [61] and the nanopore units were caged with a Cy5‐modified DNA locking strand P, resulting in “mute” inactive loaded liposome containments. Uncaging of the DNA pores with a fuel strand P’, allowed the permeation and transport of exterior Mg^2+^‐ions into the liposome reservoirs, resulting in the Mg^2+^‐ion triggered activation of the ligation of the constituents loaded in the liposomes (For the schematic assembly and gel electrophoretic assembly of the DNA origami nanopore, see Figure S1 and accompanying discussion. The characterization, optimization, and permeation properties of the integrated DNA nanopore within the liposome membrane are presented in Figures S2–S5 and accompanying discussion). This resulted in the formation of phase‐separated condensates, according to the mechanism introduced by Walther et al. [60], and displayed in Figure 2b. The inter‐hybridization of L_1_‐X duplexes bridged by M_1_ and L_2_‐Y duplexes bridged by M_2_, followed by the ligase‐induced linkage of the oligomerized subunits, yielded polymer chains of j‐tethered L_1_+X/M_1_ and j’‐tethered L_2_+Y/M_2_ repeat units, and inter‐hybridization of the polymers by the complementary j/j’ tethers yielded the phase‐separated condensates. For the preparation of functional, barcode‐modified condensates, vide infra, the constituent L_2_+Y/M_2_ (90%) and L_2_+(Y+Bs1)/M_2_ (10%) were loaded in the liposome containment, where Bs1 represents a barcode for the tailored functionality tethered to the end of Y unit in the composite, yielding the functional condensates. For gel electrophoretic characterization of ligation of the respective DNA module into the DNA polymer, see Figure S6 and accompanying discussion (For the optimization the phase‐separated formation of organelles in the liposome, a series of primary steps examining the phase separation of DNA module in a bulk homogenous solution were performed. These steps are detailed on p.S18 to p.S20 of Supporting Information and Figures S7–S9). Figure 2c depicts the confocal microscopy images corresponding to the nanopore‐triggered, ligase‐mediated formation of the fluorescein‐labeled condensates and control systems. While no condensate formation is observed in the absence of DNA nanopore component, entry (i) (only background fluorescence of the constituents), green fluorescent condensates (O_1_) are formed upon treatment of opened nanopore‐modified liposomes with Mg^2+^‐ions, entry (ii). In addition, locked nanopore units modifying the liposomes, caged with the fluorophore (Cy5) modified lock P (*λ*_em_ 633 nm), treated with Mg^2+^‐ions did not lead to condensates, while showing the background green fluorescence of the constituents in the liposomes and the red fluorescence of the locked nanopore units in the boundary domain of the liposomes, entry (iii). Unlocking the nanopores by a complementary strand P’ (displaces the fluorescent locking strand P, caging the nanopore in the liposome boundary), triggered the formation of the condensates in the liposomes, entry (iv). For the zoom‐out confocal fluorescence microscopy images of the organelles O_1_ in liposomes of different configurations, see Figure S10. Interestingly, however, we find that while using a bulk concentration of L_1_+X/M_1_ corresponding to 10 µM to prepare the liposomes, and using an appropriate calibration curve (Figure S11), the final concentration of L_1_+X/M_1_ loaded in the liposomes is only 6 µM (after purification and washing of the liposomes). Nevertheless, the Mg^2+^‐ions/ligase‐induced phase separated formation of the organelles in the liposomes proceeds effectively. Figure S12A depicts the temporal fluorescence confocal microscopy images corresponding to the formation of organelle O_1_ in the liposome, Panel I, in comparison to the formation of the condensates in the homogenous solution under similar conditions, Panel II. Figure S12B compares the rates of formation of the organelles in the liposomes versus the homogenous solution. Evidently, the rate of phase separation in the liposomes is significantly enhanced as compared to the homogenous solution, presumably due to the assembly of the condensates in the confined liposome environment. Moreover, the number of DNA origami nanopores associated with the liposome boundary should affect the rate of assembly the organelle formation in the liposomes. Indeed, Figure S13 and accompanying discussion demonstrate that as the number of pore sites generated on the liposome boundary increases, the rate of organelle formation increases.

**FIGURE 3:**
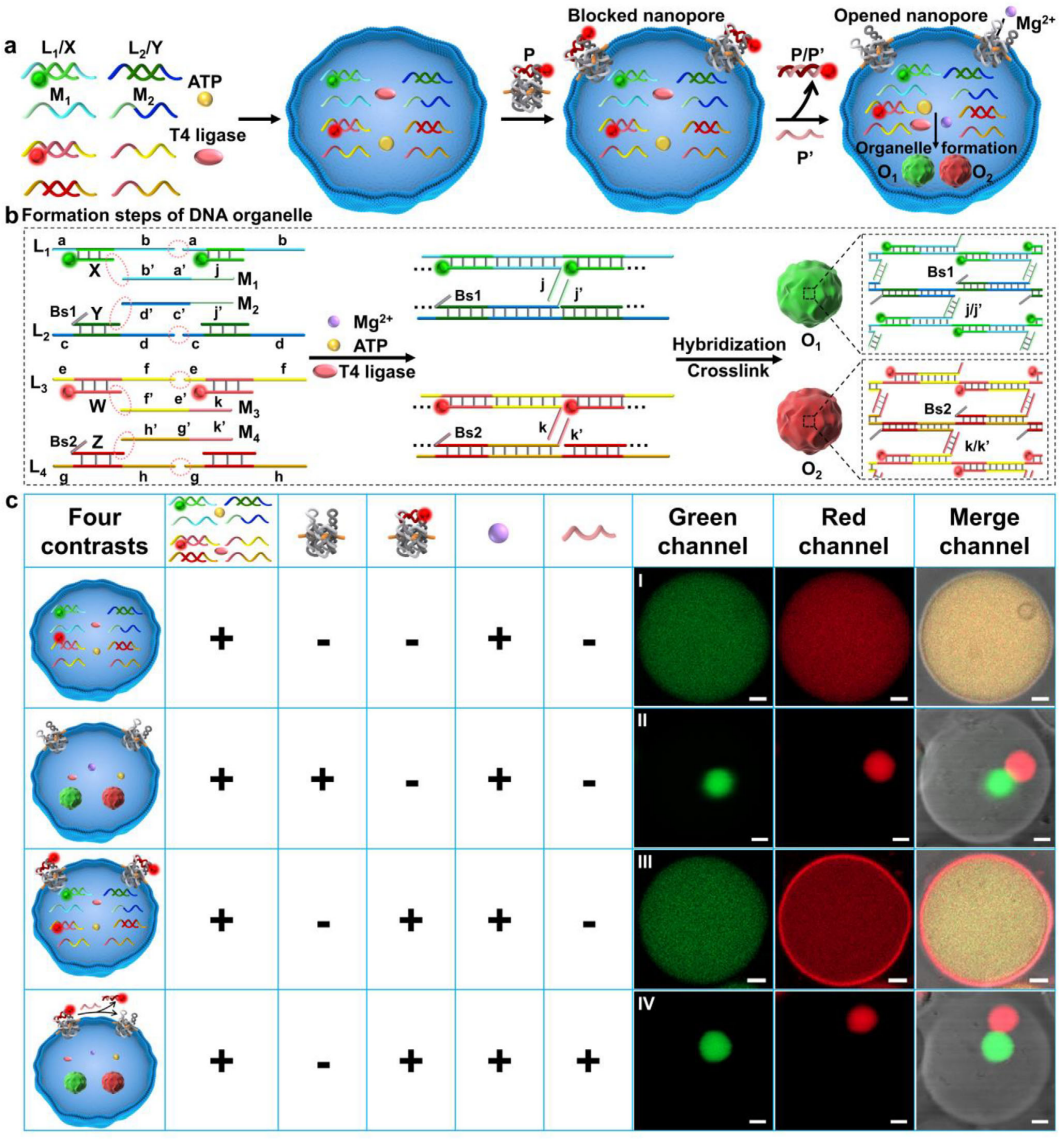
Schematic assembly and characterization of two different barcode‐functionalized organelles, O_1_ and O_2_ in the liposome carrier. (a) Integration of the pre‐engineered constituents for assembly of the two organelles in the pore‐locked liposome carrier. (b) Schematic mechanistic path for the Mg^2+^‐ion triggered formation of the two barcode‐functionalized organelles O_1_ and O_2_, upon fueled unlocking of the pores, transport of Mg^2+^‐ions through the pores, and Mg^2+^‐ions triggered ligation and guided selective hybridization of the pre‐engineered constituents into phase‐separated organelles O_1_ and O_2_. (c) Confocal fluorescence microscopy images (scale bar = 2 µm) following the phase separation of organelle O_1_ and O_2_ in the liposome carrier (and control systems).

Figure 3a,b depict the schematic formation of two different kinds of condensates as organelle mimics in the liposome containments. This was accomplished by employing two sets of constituents L_1_+X/M_1_, L_2_+Y/M_2_ (where X was labeled with FAM), and L_3_+W/M_3_, L_4_+Z/M_4_ (where W was labeled with Cy5). The fuel‐triggered uncaging of the DNA nanopores enabled, then, the Mg^2+^‐ion triggered ligation and phase separation of two distinct green O_1_ and red O_2_ condensates in the liposomes. Figure 3c displays the confocal microscopy images corresponding to the formation of the two organelles O_1_ and O_2_ and appropriate control systems. Figure 3c (entry iv) confirms the formation of two orthogonal condensates O_1_ (green fluorescence) and O_2_ (red fluorescence) upon uncaging the DNA nanopores allowing the Mg^2+^‐ion induced formation of the condensates as organelle mimics (For the zoom‐out confocal fluorescence microscopy images of the organelles O_1_ and O_2_ in liposomes of different configurations, see Figure S14 and accompanying discussion). The temporal behaviour of the green/red organelles in the liposomes is presented in Figure S15. Comparable kinetics for formation of 3–4 µm sized green/red organelles after 2 h are demonstrated.

**FIGURE 4:**
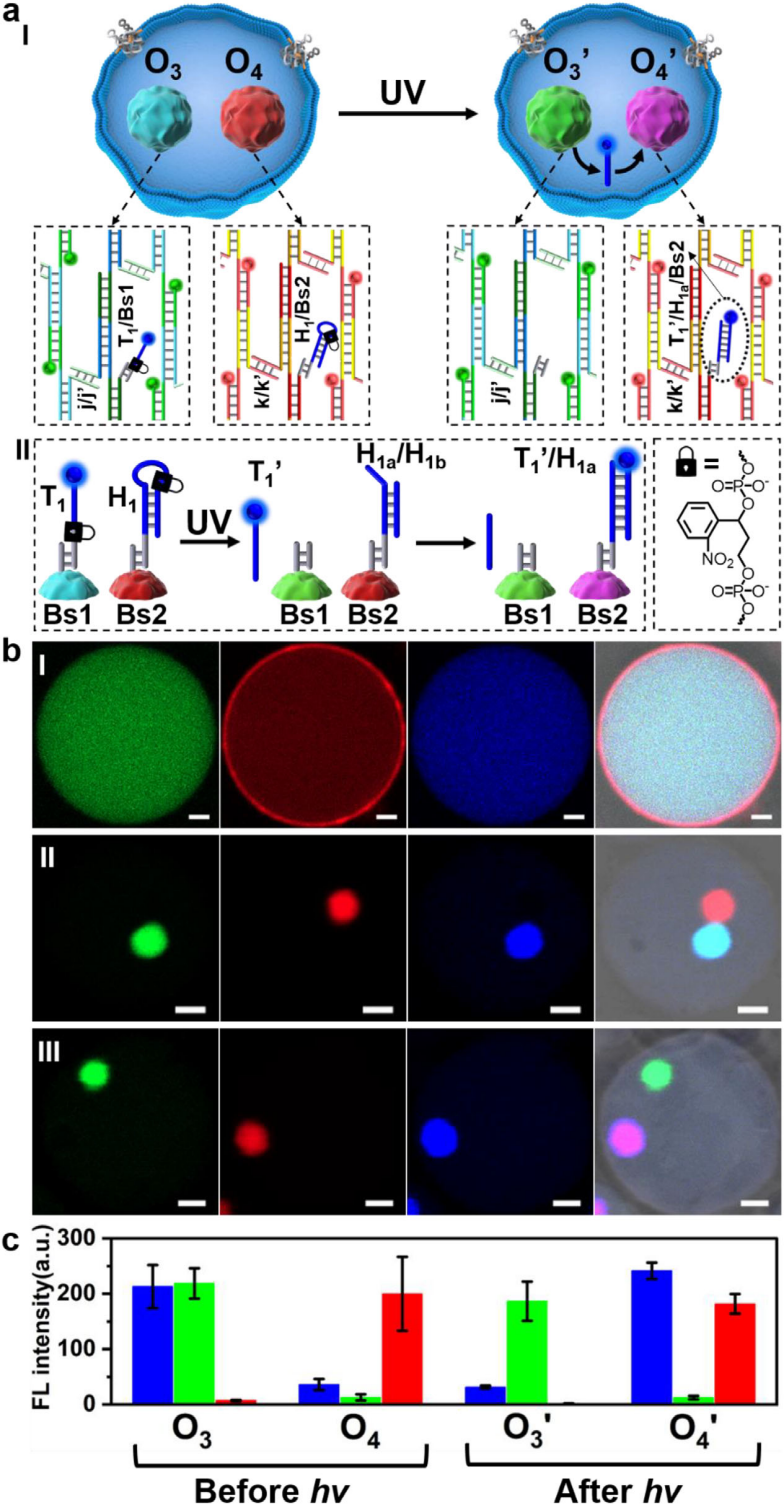
Light‐induced strand‐intercommunication and dictated reconfiguration of organelles in liposome. (a) Panel I – Assembly of two light‐responsive organelles O_3_/O_4_, undergoing light‐triggered strand‐intercommunicated reconfiguration into two new organelles O_3_’/O_4_’. Panel II – Detailed mechanistic path associated with the light‐induced generation of the information transfer strand T_1_’ stimulating the reconfiguration of O_3_/O_4_ into O_3_’/O_4_’. (b) Confocal fluorescence microscopy images (scale bar = 2 µm) probing the phase separation of the two organelles O_3_/O_4_ in the liposome containment and their light triggered reconfiguration into the O_3_’/O_4_’ organelles. Panel I – The constituent‐loaded liposome prior to the triggered opening of the pores and the Mg^2+^‐ion formation of organelles O_3_/O_4_. Panel II – After opening the pores in the liposome boundary and the Mg^2+^‐ions activation of the ligation of the constituents and self‐assembly of phase‐separated O_3_/O_4_ organelles. Panel III – After UV‐light triggered activation of organelles O_3_/O_4_ and their intercommunication reconfiguration into organelles O_3_’/O_4_’. (c) Fluorescence intensities of the constituent associated with organelles O_3_/O_4_ prior to reconfiguration and of O_3_’/O_4_’ after reconfiguration. Error bars deduced from *N* = 4 experiments.

In the next step, we synthesized functional protocells containing two light‐responsive, o‐nitrobenzyl phosphate ester [62] functionalized organelles, O_3_ and O_4_, allowing light‐triggered inter‐organelle communication and information transfer (Figure 4a). Organelle O_3_ consisted of a green fluorescent condensate formed by L_1_+X/M_1_ and L_2_+Y/M_2_, where the Y component's barcode Bs1 was hybridized with the photo‐caged, blue fluorophore‐labeled strand T_1_. Organelle O_4_ was composed of red fluorescent condensates assembled from crosslinked L_3_+W/M_3_ and L4+Z/M_4_, where the Z component's barcode Bs2 was hybridized with the photo‐caged hairpin H_1_, Panel I (For the detailed structural formation of the polymer constituents in O_3_/O_4_, see Figure S16.). Upon 365 nm light irradiation, photo‐cleavage of T_1_ and H_1_ generated the blue‐fluorescent fragment T_1_’ and the cleaved duplex H_1a_/H_1b_, Panel II. The released strand T_1_’ from O_3_ was designed to displace H_1b_ in O_4_, triggering information transfer and structural reorganization of the original organelles into O_3_’ and O_4_’. This inter‐organelle communication process, driven by the light‐activated DNA strand displacement, was confirmed by gel electrophoresis (Figure S17). Dynamic intercommunication and reconfiguration of the two organelles in liposomes were monitored via confocal fluorescence microscopy (Figure 4b). Panel I shows fluorescence images of nanopore‐locked liposomes prior to organelle formation, revealing dispersed green fluorescence from L_1_+X, red fluorescence from core of L_3_+W and rim of locked nanopores, and core‐localized blue fluorescence from T1/Bs1, indicating “mute” liposomes lacking condensed organelles. Upon unlocking the nanopores and allowing Mg^2+^ ion influx, phase‐separated condensates O_3_ and O_4_ formed (Figure 4b, Panel II). Organelle O_3_ displayed cyan fluorescence due to colocalized green and blue fluorophores, while O_4_ showed red fluorescence. These images confirmed the Mg^2+^‐ion‐induced condensation and organization of the functional organelles within the liposome protocells. Light activation of the system led to the formation of reconfigured organelles O_3_’ and O_4_’, as shown in Panel III. The resulting fluorescence profiles revealed green fluorescent O_3_’ (lacking blue T_1_’) and violet fluorescent O_4_’ (combining red and transferred blue fluorescence from T_1_’). This transformation reflected the directional strand transfer and structural reorganization following light‐triggered activation. The reconfiguration process was further quantified in Figure 4c, which shows the separated fluorescence intensities of the green, blue, and red labels in the organelles before and after light activation. Initially, O_3_ showed strong green and blue fluorescence, while O_4_ exhibited strong red fluorescence. After light activation, O_3_’ retained only green fluorescence, while O_4_’ showed strong red and blue fluorescence, confirming the intercommunication and reconfiguration processes. Additional zoom‐out fluorescence images illustrating the light‐induced intercommunication and reconfiguration of O_3_/O_4_ in liposome protocells across different configurations are provided in Figure S18. It should be noted that UV illumination of the O_3_/O_4_ organelles loaded with the respective nucleic acid constituents lacking the photocleavable protecting units did not have any structural perturbation on the integrity of the organelles (Figure S19).

Furthermore, the light‐triggered intercommunication between organelles O_3_ and O_4_ in the liposome assembly has important confined media advantages, emulating native cells. Figure S20A depicts the temporal intercommunication efficiency of the O_3_/O_4_ organelle transition to the O_3_’/O_4_’ state demonstrating a ca. 86% transition yield within 2 min. In contrast, Figure S20B presents the intercommunication efficiency of O_3_/O_4_ to O_3_’/O_4_’ in a homogenous solution. Evidently, the transition yields correspond to ca. 30% after 30 min. That is, intercommunication between the organelles in the liposome assembly is ca. 3‐fold enhanced in the confined environment. It should be noted that in a previous study, switchable light‐induced strand transfer transitions between phase‐separated coacervates in microfluidic‐generated aquous droplets (75 µm) has been reported [46], yet the effect of spatial confinement was not demonstrated, presumably due to the 5‐fold increased size of the droplet as compared to the liposomes used in our study.

**FIGURE 5:**
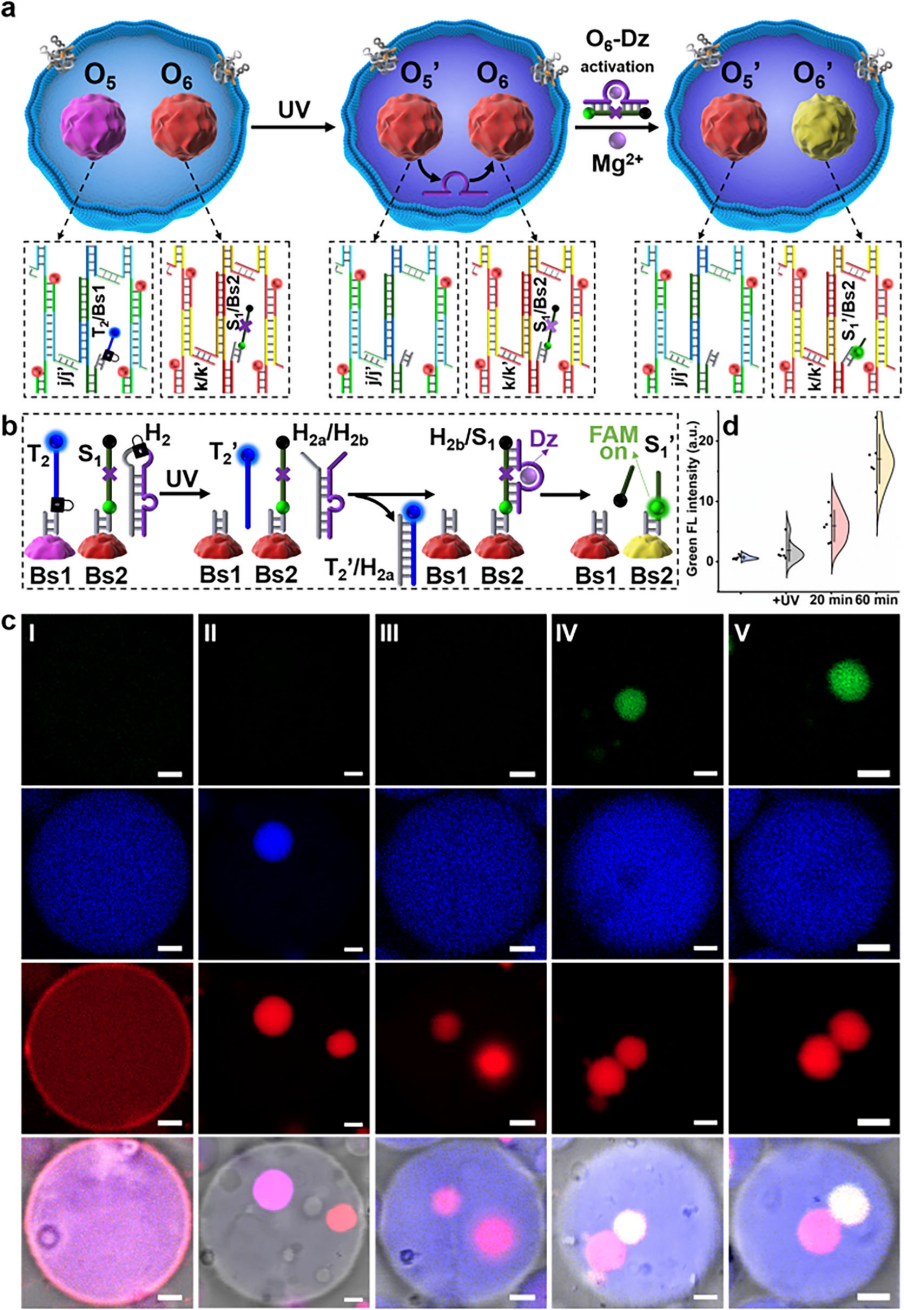
Light‐induced, DNAzyme‐mediated, reconfiguration of two organelles in the liposome assembly. (a) Schematic composition of two phase‐separated organelles O_5_/O_6_, their intermediate light‐induced generation of organelles O_5_’/O_6_ and the subsequent DNAzyme‐mediated reconfiguration of the organelles into the O_5_’/O_6_’ state. (b) Stepwise mechanistic display of the steps involved with the light‐induced activation of organelles O_5_/O_6_, in the presence of hairpin H_2_, to undergo DNAzyme‐mediated reconfiguration to the O_5_’/O_6_’ state. (c) Confocal fluorescence microscopy images (scale bar = 2 µm) corresponding to: Panel I – the constituents in the pore‐locked liposomes, prior to phase separation; Panel II – after unlocking the pores and inducing the phase‐separated organelles O_5_/O_6_; Panels III–V – after light‐induced activation of organelles O_5_/O_6_ and recording at time‐intervals the fluorescence features of the liposome, upon dynamic reconfiguration into the O_5_’/O_6_’ state (Panel III – after 0 min, Panel IV – after 20 min, Panel V – after 60 min). (d) Time‐dependent integrated fluorescence intensities of the green FAM fluorescence upon dynamic formation of organelle O_6_’.

The successful construction of liposome protocells containing two distinct phase‐separated DNA condensates was further employed to assemble functional organelles O_5_ and O_6_, enabling light‐triggered inter‐organelle communication and activation of a catalytic DNAzyme in O_6_ (Figure 5). This system emulates biological information transfer between source and drain organelles evolving catalytic functions. Figure 5a,b schematically illustrates the composition of organelles O_5_ and O_6_ and the communication mechanism leading to the DNAzyme activity in O_6_. Organelle O_5_ is formed via j/j’ crosslinking of two ligated polymer chains. One chain contains subunits L_1_+X (red fluorophore‐labeled) bridged by M_1_ (modified with j), while the second chain comprises L_2_+(Y+Bs1) subunits bridged by M_2_ (modified with j’). A blue‐fluorescent, photo‐caged DNA strand T_2_ is hybridized with barcode Bs1. The hybrid yields O_5_ with violet fluorescence (blue + red). Organelle O_6_ is constructed by k/k’ crosslinking of two polymer chains. One includes M_3_‐bridged L_3_+W (red fluorophore‐labeled) with tether k, and the other has M_4_‐bridged L_4_+(Z+Bs2) with a functional strand S_1_ hybridized to Bs2. Strand S_1_ is modified with a ribonucleobase (rA) and a FAM/BHQ‐1 fluorophore‐quencher pair, resulting in quenched green fluorescence. O_6_, therefore, exhibits red fluorescence. Thus, O_5_ and O_6_ organelles reveal violet and red fluorescence, respectively, inside the liposome (For a detailed structural formation of the polymer constituents in O_5_/O_6_, see Figure S21.). An additional photo‐caged hairpin H_2_, containing a “caged” inactive Mg^2+^‐dependent DNAzyme sequence, is included in the liposome core. Upon 365 nm light irradiation, photochemical uncaging of T_2_ and H_2_ releases the blue‐fluorescent strand T_2_’ and forms duplex H_2a_/H_2b_ (Figure 5b). Strand T_2_^'^ displaces H_2a_/H_2b_, releasing strand Dz (H_2b_), the active DNAzyme. Dz migrates to organelle O_6_ and cleaves the S_1_ substrate, releasing the quencher‐modified DNA fragments, triggering the green FAM fluorescence. This catalytic reaction marks the successful transfer of the information strand Dz from the O_5_ to O_6_, resulting in the reconfiguration of O_5_/O_6_ into O_5_’ (revealing red fluorescence) and O_6_’ (showing yellow fluorescence from overlapping green and red fluorophores). The inter‐organelle communication mechanism, followed by emergent DNAzyme activity, was confirmed by gel electrophoresis and DNAzyme cleavage of substrate (Figure S22). The sequential fluorescence features conforming the transition of O_5_/O_6_ into the O_5_’/O_6_’ organelles are displayed in Figure 5c, probing the fluorescence of the organelles through the respective fluorescence confocal microscopy channels. Panel I shows the initial fluorescence of the liposome‐loaded contents. Panel II reveals Mg^2^^+^‐induced organelle condensation with violet fluorescent O_5_ and red fluorescent O_6_. Panels III–V show time‐lapse images of organelle fluorescence after photochemical activation: Panel III (0 min), Panel IV (20 min), and Panel V (60 min). After a time‐interval of 60 min, O_5_' retains red fluorescence, while O_6_’ exhibits yellow fluorescence (green + red), confirming successful DNAzyme activation. Residual bulk blue fluorescence originates from the released strands T_2_'/H_2a_. Additional zoom‐out fluorescence images showing the light‐induced intercommunication and DNAzyme‐mediated reconfiguration of O_5_/O_6_ in liposomes are provided in Figure S23. Figure 5d presents the time‐dependent increase in green fluorescence intensities, confirming the inter‐organelle communication pathway and catalytic transformation of O_6_ into O_6_’.

**FIGURE 6:**
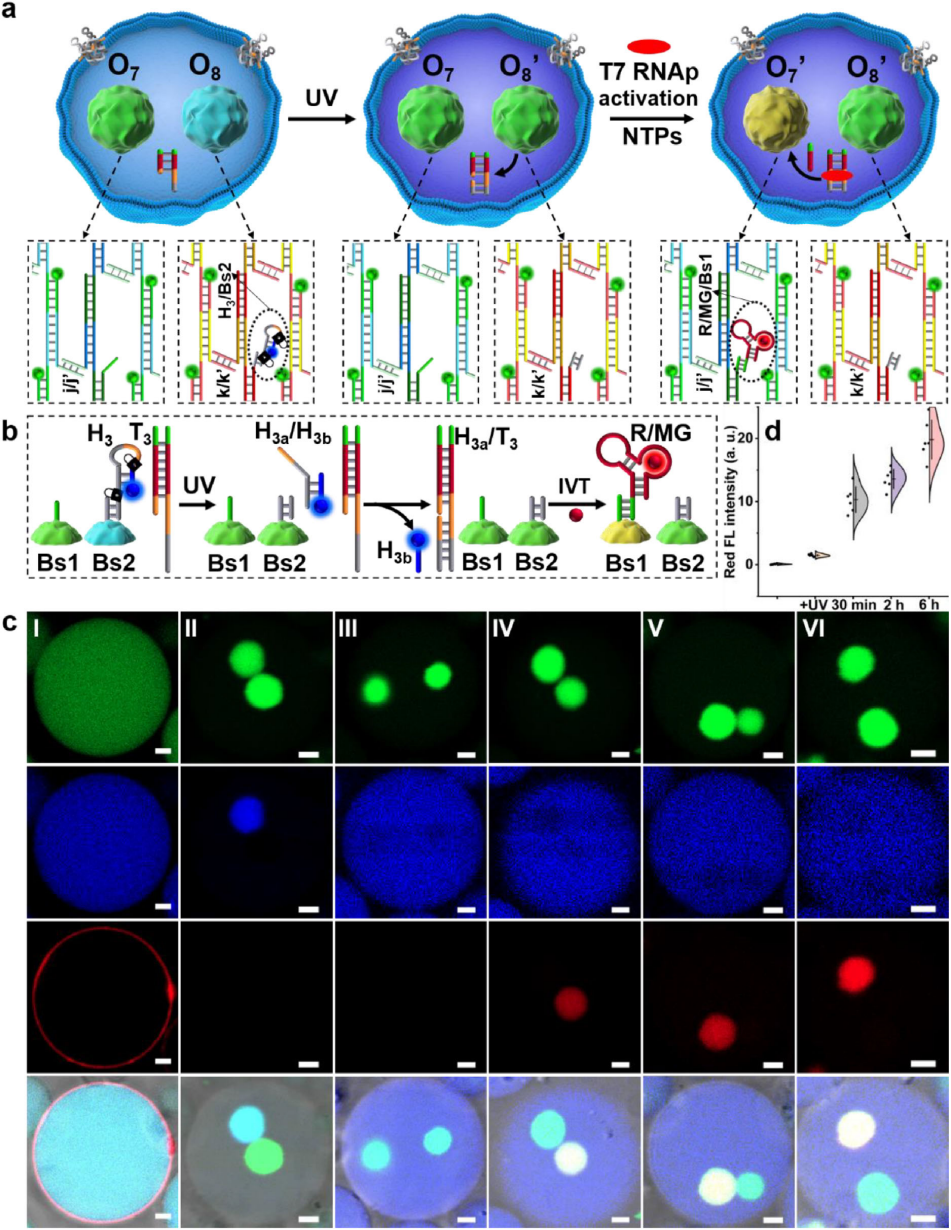
Light‐induced, transcription machinery‐guided, reconfiguration of two organelles in the liposome. (a) Schematic composition of two phase‐separated organelles O_7_/O_8_, undergoing light‐activated transition to state O_7_/O_8_’ that in the presence of an auxiliary transcription machinery (template H_3a_/T_3_ and T7 RNAp/NTPs) reconfigure in the O_7_’/O_8_’ organelle state. (b) Mechanistic display of the light‐activated and transcription machinery‐guided dynamic reconfiguration of organelles O_7_/O_8_ to O_7_’/O_8_’ state. (c) Confocal fluorescence microscopy images (scale bar = 2 µm) corresponding to: Panel I‐the constituents in the pore‐locked liposomes, prior to phase separation; Panel II‐after unlocking the pores and Mg^2+^‐ions induced phase‐separated organelles O_7_/O_8_; Panel III‐Panel VI‐after light‐induced activation of organelles O_7_/O_8_ and recording at time‐intervals the fluorescence features of the liposome, upon dynamic reconfiguration into the O_7_’/O_8_’ state (Panel III – after 0 min, Panel IV – after 30 min, Panel V – after 2 h, Panel VI – after 6 h). (d) Time‐dependent integrated fluorescence intensities of the red MG‐RNA aptamer fluorescence upon dynamic formation of organelle O_7_’.

In a further example, intercommunication between two organelles O_7_ and O_8_ in the liposomes is accomplished by a transcription machinery guiding the structural reconfiguration of the two organelles (Figure 6a,b). Organelle O_7_ comprises two j/j’‐crosslinked polymer chains: one formed by M_1_‐bridged L_1_+X duplex units (X labeled with green FAM), and the other by M_2_‐bridged L_2_+Y duplex units, with Y functionalized with barcode tethers Bs1. Organelle O_8_ consists of k/k’‐crosslinked polymer chains: one chain has M_3_‐bridged L_3_+W duplex subunits (W is FAM‐labeled), and the other chain includes M_4_‐bridged L_4_+Z duplex units, where Z is functionalized with barcode tethers Bs2. A blue‐fluorescent hairpin H_3_, caged with two photoresponsive o‐nitrobenzyl phosphate ester groups, is hybridized with the Bs2 units on O_8_ (For detailed definition of the polymer chain constituents in O_7_/O_8_, see Figure S24.). Structurally, O_7_ exhibits green fluorescence, while O_8_ displays cyan fluorescence (green + blue). The bulk liposome also includes an inactive transcription template T_3_, T7 RNA polymerase (T7 RNAp), nucleoside triphosphates (NTPs), and non‐fluorescent malachite green (MG). Figure 6b shows the light‐activated transcription pathway enabling information transfer between organelles and their structural reconfiguration. Upon 365 nm irradiation, the caged H_3_ linked to O_8_ is cleaved, releasing the duplex H_3a_/H_3b_ into the liposome volume. H_3b_ is designed to be displaced by template T_3_, resulting in the formation of an active H_3a_/T_3_ transcription template and the release of the blue‐fluorescent H_3b_. This triggers the transcriptional machinery to synthesize the RNA product R, specifically, an MG‐binding RNA aptamer that is pre‐engineered by the transcription template to include a tether sequence complementary to the Bs1 barcode on O_7_. The resulting fluorescent MG/RNA aptamer complex (*λ*_em_ = 665 nm, red) hybridizes with Bs1 associated with O_7_. (For a control experiment confirming the light‐induced cleavage of the H_3_, activating the transcription machinery synthesizing the MG RNA aptamer in a homogeneous buffer solution, see Figure S25 and accompanying discussion.) Thus, the light‐induced cleavage of H_3_ in organelle O_8_ activates operation of the transcription machinery synthesizing the MG RNA aptamer sequence, acting as information transfer strand that binds to O_7_. Figure 6c shows the temporal confocal fluorescence microscopy images of the light‐induced, transcription machinery‐guided dynamic inter‐organelle communication. Panel I presents the initial state of liposomes loaded with constituents of green (X + W), blue (H_3_), and red (nanopore locking strand), using the respective fluorescence channels, resulting in the overlayed cyan fluorescent liposome interior, surrounded by red boundary fluorescence. Panel II depicts the liposomes after nanopore‐unlocking and Mg^2^^+^‐induced phase‐separated transformation of the organelles. Distinct green fluorescence marks O_7_, while O_8_ shows cyan fluorescence, and the red fluorescence signal associated with liposome boundary is lost, confirming nanopore‐unlocking. Panels III–VI illustrate fluorescence changes over 6 h post‐photoactivation. Immediately after light exposure, blue fluorescence dissipates from O_8_ and redistributes into the liposome volume, indicating release of H_3a_/H_3b_, and only green‐fluorescent organelles corresponding to O_7_ and the reconfigured O_8_’ (no longer binds H_3_ and appears green), are observed. As time progresses, transcription in the liposome bulk yields the MG‐aptamer RNA, which binds to O_7_ and transforms it into O_7_’, exhibiting overlayed yellow (green + red) fluorescence. Meanwhile, O_8_’ reveals green fluorescence, and H_3b_ maintains a diffuse blue signal in the bulk. These observations confirm the light‐triggered cleavage of H_3_, release of H_3a_/H_3b_, and transcriptional activation in the liposome core, resulting in dynamic structural reconfiguration of O_7_ into O_7_’ (yellow) while O_8_ transforms into O_8_’ (green). Additional zoom‐out fluorescence images showing the light‐induced intercommunication and transcription machinery‐mediated reconfiguration of O_7_/O_8_ in liposomes are provided in Figure S26. Figure 6d quantifies the red fluorescence intensity over time, corresponding to MG‐aptamer hybridization with O_7_’. Thus, the data validate initial organelle phase separation, photoinduced reconfiguration of O_8_ into O_8_’, and transcription‐guided structural transition of O_7_ into O_7_’.

## 3 Conclusion

Assembly of synthetic intercommunicating functional phase‐separated organelles in cell‐like containments is of key significance for developing protocells emulating native cell functions. The study introduced a versatile method to assemble oligonucleotide barcode‐tethered organelles in DNA nanopore‐modified liposome protocells. The transport of Mg^2+^‐ions across the nanopores triggered the ligation of pre‐programmed constituents embedded in the liposome protocells forming polymer nucleic acid chains that inter‐crosslinked to form phase‐separated barcode‐tethered organelles. By loading two different sets of pre‐programmed constituents in the liposome protocells, assembly of two different organelles in the liposome protocells was demonstrated. Through the hybridization of o‐nitrobenzyl phosphate ester‐caged photoresponsive auxiliary nucleic acids and other pre‐tailored auxiliary oligonucleotides with the barcode tethers, functional, light‐responsive organelles were assembled. Light‐triggered uncaging of the respective constituents resulted in the release of functional strands acting as inter‐organelle communication strands dictating structural reconfiguration of the organelles. By pre‐engineering the barcode‐hybridized oligonucleotides, the photo‐triggered intercommunication information strands activated a DNAzyme catalytic or an intra‐liposome transcription machinery, resulting in reconfiguration of the organelles composition. Beyond advancing the area of System Chemistry, the method introduced in our study provides innovative tools to develop protocells.The significant accomplishment of the present study are reflected by the programmability of the functional organelles that signaled intercommunication between the organelles leading to structural and catalytic outputs emulating native cell functions. The systems provide a fundamental concept to establish functional communication between synthetic organelles. By the integration of other selective nanopore units into the liposome membranes, transport of ligand through the nanopores, particularly ligand recognized by aptamers embedded in the organelles, aptamer/ligand‐intercommunication between the organelles and emerging organelle functionalities may be envisaged. Furthermore, the release of pre‐engineered oligonucleotide strands by the DNAzyme or transcription could provide means to induce subsequent ligation and phase separation of a third kind of organelle condensates, intercommunicating with the parent organelles. Moreover, recent studies reported on the fusion of loaded liposomes with native cell and the delivery of the liposome loads into the cells [63, 64]. These methods could be adapted to deliver synthetic organelles, thereby controlling cell functions (For the stability of the organelle‐loaded liposome carrier in cell culture medium, see Figure S27).

## Author Contributions

**Huiying Xue**: data curation, methodology, validation, conceptualization, formal analysis, investigation, writing – original draft, writing – review and editing, visualization. **Yunlong Qin**: methodology, validation, writing – review and editing, writing – original draft, data curation, visualization. **Yichen Han**: writing – review and editing, data curation, visualization. **Shijun Xu**: data curation, methodology. **Itamar Willner**: conceptualization, methodology, writing – original draft, writing – review and editing, project administration, supervision, funding acquisition, visualization, validation. **Fan Xia**: conceptualization, methodology, resources, project administration, writing – review and editing, writing – original draft, funding acquisition. **Fujian Huang**: funding acquisition, writing – review and editing, writing – original draft, project administration, resources, supervision, validation, methodology, conceptualization

## Conflicts of Interest

The authors declare no conflicts of interest.

## Supporting information




**Supporting File**: Experimental section, detailed DNA sequences, supporting figures and accompanying discussions.

## Data Availability

The data that support the findings of this study are available from the corresponding author upon reasonable request.
