# Supplementary material for: Photoactivated Signaling Networks using DNA‐Based Synthetic Organelles as Biomimetic Protocells
Source: Angew Chem Int Ed Engl. 2026 May 1;65(25):e4889049. doi: 10.1002/anie.4889049 (PMC13266963; doi:10.1002/anie.4889049)
Supplement: Supplementary file 1 — Supporting File: Experimental section, detailed DNA sequences, supporting figures and accompanying discussions. [file ANIE-65-e4889049-s001.docx]

Supporting information for

**Photoactivated Signaling Networks in DNA-Based Synthetic Organelles as Biomimetic Protocells.**

Huiying Xue^[a],#^, Yunlong Qin^[b],#^, Yichen Han^[b]^, Shijun Xu^[a]^, Itamar Willner*^[b]^, Fan Xia*^[a]^, and Fujian Huang*^[a]^

[a] H. Xue, S. Xu, Prof. F. Xia, Prof. F. Huang
State Key Laboratory of Geomicrobiology and Environmental Changes, Faculty of Materials Science and Chemistry
China University of Geosciences
Wuhan 430074, China
Email: xiafan@cug.edu.cn; huangfj@cug.edu.cn

[b] Y. Qin, Y. Han, Prof. I. Willner
The Institute of Chemistry
The Hebrew University of Jerusalem
Jerusalem 91904, Israel
E-mail: [itamar.willner@mail.huji.ac.il](mailto:itamar.willner@mail.huji.ac.il)

#These authors contributed equally.

**Experimental Section**

**1. Materials and Characterization**

Tris(hydroxymethyl)aminomethane (Tris), chloroform, Sucrose, D-(+)-Glucose, KCl, NaCl, HCl, MgCl_2_, 1,4-Dithiothreitol (DTT), 1,2-dioleoyl-sn-glycero-3-phosphorylcholine (DOPC), Dipalmitoyl Phosphatidylcholineand (DPPC), mineral oil and cholesterol were purchased from Aladdin Scientific Corp. 10×PBS and 1-palmitoyl-2-oleoyl-sn-glycero-3-phosphocholine (POPC) were purchased from Shanghai yuanye Bio-Technology Co., Ltd. DSPE-PEG2000 and Malachite green (MG) were purchased from Shanghai Macklin Biochemical Technology Co. Ltd. T4 DNA Ligase (30 WU/μL) was purchased from Thermo Fisher Scientific. T7 RNA Polymerase (50,000 U/ml) was purchased from New England Biolabs. ATP (100 mM), NTP Mix (25 mM each) was purchased from Beyotime Biotech Inc**.** All chemical reagents used in the experiments were analytically pure without further purification. All DNA sequences were purchased from Hippo Biotech (Beijing, China) with HPLC purification, and dissolved in TE buffer (10 mM Tris-HCl, 1 mM EDTA, pH 8.0). Detailed DNA sequences are shown in Table S1, S2 and S3. All DNA with a hairpin structure is used after a single rapid annealing in 1×PBS buffer. During the whole experiments, ultrapure water (18.25 mΩ cm) was used by Heal Force Water Purification Systems (Shanghai, China).

Confocal fluorescence microscopy imaging was conducted using a Zeiss LSM 880 confocal laser-scanning microscope (CLSM) and Zeiss LSM 980 confocal laser-scanning microscope (Carl Zeiss). The excitation of FAM, Cy3 and Cy5 fluorophores was performed using lasers with wavelengths of 488 nm, 543 nm, and 633 nm, respectively. Gel electrophoresis experiment was performed using Liuyi DYCZ-24FN and the gel image was acquired using a Tanon imaging system (Tanon 5200 Multi).

**2. Construction of DNA nanostructures.**

Nanopore was heated to 95 degrees for two minutes and then slowly cooled to room temperature on a metal bath in 1×TAE/Mg^2+^ buffer (40mM Tris, 20mM acetic acid, 1mM EDTA, pH=7.4, 12.5 mM Mg^2+^). The DNA sequence used for DNA nanopore is shown in Supplementary Table S1, also see Figure S1.

**3. Polyacrylamide gel electrophoresis (Native)**

The assembly of DNA origami pore and DNA molecule intercommunication in organelles, were characterized by gel electrophoresis, where corresponding DNA samples (10 µL, 1 µM) were mixed with 6× gel loading dye (2 µL) in 1× Tris-acetate-EDTA/Mg^2+^ buffer (40 mM Tris-acetate, 12.5 mM magnesium acetate, and 1 mM EDTA, pH 8.0). The samples were loaded and electrophorized at 110 V for 60 min. Afterwards, the polyacrylamide gel was stained with Gel-red DNA staining dye for 10 min, washed three times, and imaged by a molecular imager using UV light.

**4. Preparation of giant unilamellar vesicles (GUVs) as liposome protocell containment.**

**4.1 Electroformation method and the assessment of membrane-integrated DNA nanopore.**

Liposomes were prepared using the electroformation method with the Vesicle Prep Pro (Nanion Technologies GmbH, Munich, Germany). A lipid-cholesterol mixture (20 μL, containing 5 mM DPPC and 2 mM cholesterol) was prepared, and then evenly spread on the surface of indium tin oxide (ITO)-coated glass. The lipid-coated ITO slides were obtained by desiccation of 15 min to remove the chloroform. An electroformation chamber was constructed by sandwiching a rubber gasket between the lipid-coated ITO slide and another ITO slide, and filled with 300 mM sucrose. Liposomes were formed by applying alternating current between two ITO slides (3 V, 5 Hz) at 50°C for 150 min. Afterwards, Liposomes were gently collected and kept at 4 °C within one week for subsequent experiments.

The nanopore was anchored into the Liposome membrane via hydrophobic interaction. In detail, 2 μL of the Liposomes were mixed with DNA nanopore (300 nM unless otherwise stated) in 1×PBS buffer supplemented with 10 mM Mg^2+^, and the final volume is 10 μL. After incubation for 15 min, R6G or unlocking DNA strand P’ was added according to the conditions in Figure S1, and then imaging was performed by CLSM. The nanopore-integrated liposomes here were used to confirm the permeability of the liposome in a pore-locked or pore-unlocked state, and optimize the surface coverage of nanopore units, see Figure S2-S5.

**4.2 Emulsion method**

DOPC, POPC, cholesterol, and DSPE-PEG2000 were dissolved in chloroform in a glass tube (in a molar ratio of 4:4:1.9:0.1, totally 15 mM). DSPE-PEG2000 was added to prevent nonspecific adhesion between proteins and the lipid membrane. The lipid mixture in chloroform was bubbled with nitrogen and subsequently dried under vacuum. Then, mineral oil was added to the lipid mixture, to reach a final lipid concentration of 3 mM in the oil phase. The lipid mixture in mineral oil was then vortexed and sonicated at 70°C for at least 60 min.

The internal solution of liposome includes buffer, sucrose, and different DNA/protein loads in each experiment (c.f. section 6). The outer solution of liposome comprises of buffer and glucose, and the osmotic pressure of the vesicles was maintained by changing the glucose concentration.

To prepare loaded liposomes, 20 μL of the inner solution was added to 300 μL of the lipid mixture in mineral oil in a glass tube and then vortexed for 40 s to obtain a water-in-oil emulsion. The emulsion oil solution was gently transferred onto 300 μL of the outer solution in a test tube and subsequently centrifuged at 14000 g at 4 °C for 1 min. The precipitated inner solution-loaded liposomes were collected from the bottom and then re-dispersed in outer solution by pipetting.

Except for experiments in Figure S2-S5, liposomes in other experiments were synthesized by this method.

**5. Synthesis of phase-separated DNA microdroplet condensates in bulk solution.**

The double stranded DNA modules in the study were prepared by annealing the constituents (at 1:1 ratio) in 1×PBS at 95 °C and rapidly cooling to 4 °C.

Typical experiments were conducted in 1×TDA/Mg^2+^ buffer (30 mM Tris-HCl, 10 mM MgCl_2_, 10 mM DTT, 1 mM ATP, pH 7.8) at 37 °C containing 0.01 mM L1+X, 0.012 mM M1, 0.01 mM L2+Y, 0.012 mM M2, 0.92 WU/µL T4 DNA ligase under orbital shaking at 80 rpm. The reaction solution was sealed in a well of an CLSM imaging plate by a layer of hexadecane to avoid the possible sample evaporation and volume changes. The samples of phase-separated microdroplets were imaged by CLSM (Results in Figure S7-S10).

**6.** **Synthesis of phase-separated DNA organelles in liposome protocells.**

**6.1 Assembly of green organelle O_1_ in liposome protocells.**

The liposomes’ inner solution for this experiment is 10 µM L_1_+X, 12 µM M_1_, 9 µM L_2_+Y, 10 µM M_2_ (doped with 1 µM L_2_+Y+Bs1/M_2_ ligated polymer), 300mM sucrose and 0.92 WU/µL T4 DNA ligase in liposome buffer1 (30mM Tris-HCl, 50 mM KCl, 10mM DTT, 1mM ATP, pH 7.8). After the liposomes was synthesized, 300 nM DNA nanopores and 10 mM Mg^2+^ were added, then incubated at 37 °C for two hours and imaged by CLSM.

L_2_+Y+Bs1/M_2_ ligated polymer was prepared in 1× TDA/Mg^2+^ buffer at 37 °C containing 20 µM L_2_+Y+Bs1, 24 µM M_2_, 0.92 WU/µL T4 DNA ligase, under orbital shaking at 80 rpm for overnight. Afterwards, the remaining Mg^2+^ was washed off by centrifugation with a 3k-molecular-weight cutoff spin filter, using buffer (30mM Tris-HCl, 50 mM NaCl, 10 mM DTT, 1 mM ATP, pH 7.8).

**6.2 Assembly of green and red organelles O_1_/O_2_ in liposome protocells.**

The liposomes’ inner solution for this experiment is 10 µM L_1_+X, 12 µM M_1_, 9 µM L_2_+Y, 10 µM M_2_ (doped with 1 µM L_2_+Y+Bs1/M_2_ ligated polymer), 10 µM L_3_+W, 12 µM M_3_, 9 µM L_4_+Z, 10 µM M_4_ (doped with 1 µM L_4_+Z+Bs2/M_4_ ligated polymer), 300mM sucrose and 0.92 WU/µL T4 DNA ligase in liposome buffer1 (30mM Tris-HCl, 50 mM KCl, 10mM DTT, 1mM ATP, pH 7.8). After the liposomes was synthesized, 300 nM DNA nanopores and 10 mM Mg^2+^ were added, then incubated at 37 °C for two hours and imaged by CLSM.

**6.3 Light-triggered information transfer and reconfiguration of organelles O_3_/O_4_ in liposome protocells.**

The liposomes’ inner solution for this experiment is 10 µM L_1_+X, 12 µM M_1_, 9 µM L_2_+Y, 10 µM M_2_ (doped with 1 µM L_2_+Y+Bs1/M_2_ ligated polymer), 10 µM L_3_+W, 12 µM M_3_, 9 µM L_4_+Z, 10 µM M_4_ (doped with 1 µM L_4_+Z+Bs2/M_4_ ligated polymer), 1 µM T_1_, 1 µM H_1_, 300mM sucrose and 0.92 WU/µL T4 DNA ligase in liposome buffer1. 300 nM DNA nanopores and 10 mM Mg^2+^ were added in liposomes, then incubated at 37 °C for two hours and applied UV for 5 minutes.

**6.4 Light-triggered, evolved DNAzyme-dictated reconfiguration of organelles O_5_/O_6_ in liposome protocells.**

The liposomes’ inner solution for this experiment is 10 µM L_1_+X, 12 µM M_1_, 9 µM L_2_+Y, 10 µM M_2_ (doped with 1 µM L_2_+Y+Bs1/M_2_ ligated polymer), 10 µM L_3_+W, 12 µM M_3_, 9 µM L_4_+Z, 10 µM M_4_ (doped with 1 µM L_4_+Z+Bs2/M_4_ ligated polymer), 1 µM T_2_, 1 µM S_1_, 1 µM H_2_, 300mM sucrose and 0.92 WU/µL T4 DNA ligase in liposome buffer1. 300 nM DNA nanopores and 10 mM Mg^2+^ were added in liposomes, then incubated at 37 °C for two hours and applied UV for 5 minutes. At different time intervals, CLSM was applied to characterize the dynamic organelles reconfiguration.

**6.5 Light-triggered, evolved transcription machinery-dictated reconfiguration of organelles O_7_/O_8_ in liposome protocells..**

The dsDNA transcription template were quickly annealed from Non-template and Incomplete template with the same stoichiometry from 95 to 4°C in 1×PBS. The liposomes’ inner solution for this experiment is 10 µM L_1_+X, 12 µM M_1_, 9 µM L_2_+Y, 10 µM M_2_ (doped with 1 µM L_2_+Y+Bs1/M_2_ ligated polymer), 10 µM L_3_+W, 12 µM M_3_, 9 µM L_4_+Z, 10 µM M_4_ (doped with 1 µM L_4_+Z+Bs2/M_4_ ligated polymer), 1 µM H_3_, 0.5 µM T_3_, 300 mM sucrose, 1 mM NTP, 2 U/µL T7 RNA Polymerase, 0.92 WU/µL T4 DNA ligase in liposome buffer2 (30mM Tris-HCl, 50 mM KCl, 1mM ATP, pH 7.8). 300 nM DNA nanopores and 10 mM Mg^2+^ were added in liposomes, then incubated at 37 °C for two hours. 50 µM MG was added to the liposomes and then irradiated with UV for 5 minutes. At different time intervals, CLSM was applied to characterize the dynamic organelles reconfiguration.

**Table S1: Oligonucleotide sequences of DNA origami nanopore.**

| Name | Sequence (5'-3') |
| --- | --- |
| P1 | AGCGAACGTGGATTTTGTCCGACATCGGCAAGCTCCCTTTTTCGACTATT-chol |
| P2 | CCGATGTCGGACTTCCCAGGTTTTTACTCCGCTTACACGATCTTCGCCTGCTGGGTTTTGGGAGCTTG |
| P3 | CGAAGATCGTGTTTTTCCACAGTTGATTGCCCTTCACTTTTCCCAGCAGG-chol |
| P4 | AATCAACTGTGGTTTTTCTCACTGGTGATTAGAATGCTTTTGTGAAGGGC-chol |
| P5 | TCACCAGTGAGATTCCCAGGTTTTTACTCCGCTTTGTCGTACCAGGTGCATGGATTTTTGCATTCTAA |
| P6 | CCTGGTACGACATTTTTCCACGTTCGCTAATAGTCGATTTTATCCATGCA-chol |
| P | Cy5-ACCTGGGGTGGTATTGCGGAGTGAGGTTTTACCTGGGGTGGTATTGCGG |
| P’ | CCGCAATACCACCCCAGGTAAAACCTCACTCCGCAATACCACCCCAGGT |

**Table S2: Oligonucleotide sequences of** **DNA microdroplet condensates skeleton.**

| Name | Sequence (5'-3') |
| --- | --- |
| L_1_ | Phos-GATTAGAGACCGTACCTACATATAGCTACTGATACTCT |
| X | FAM-AGCTATATGTAGGTACGGTCTCT |
| M_1_ | Phos-AATCAGAGTATCTTTTTCGAATAGAGG |
| L_2_ | Phos-ATGAAGAGACCGTACCTACATATAGCTACTACTTGATA |
| Y | AGCTATATGTAGGTACGGTCTCT |
| M_2_ | Phos-TCATTATCAAGTTTTTTCCTCTATTCG |
| L_3_ | Phos-GTCAAGAGACCGTACCTACATATAGCTACTCATTCCAA |
| W | Cy5-AGCTATATGTAGGTACGGTCTCT |
| M_3_ | Phos-TGACTTGGAATGTTTTTCTGTGACTGT |
| L_4_ | Phos-CTTAAGAGACCGTACCTACATATAGCTACTCCTATGTG |
| Z | AGCTATATGTAGGTACGGTCTCT |
| M_4_ | Phos-TAAGCACATAGGTTTTTACAGTCACAG |

**Table S3: Oligonucleotide sequences** **for dynamic organelles reocnfiguration.**

| Y+Bs1 | TGTCTTCAGGATAGAAGCTATATGTAGGTACGGTCTCT |
| --- | --- |
| Z+Bs2 | GACTGTACGAGTTCAAGCTATATGTAGGTACGGTCTCT |
| T_1_ | Cy3-GTGTGATGAGTGTGATTGCTTCAC/PC-Linker/TCTATCCTGAAGACA |
| H_1_ | GTGTGATGAGTGTGATTG/PC-Linker /GTGAAGCAATCACACTCATCACACTGAACTCGTACAGTC |
| T_2_ | Cy3-GTCCAGGCAAGCTACAAAGATGTGCGGAGCT/PC-Linker/TCTATCCTGAAGACA |
| S_1_ | BHQ1-CATGCACA/rG/TGGACCAG/6-FAM/TTTTTGAACTCGTACAGTC |
| H_2_ | CTGGTCCAGGCAAGCTACAACGATGTGCATG/PC-Linker/AGCTCCGCACATCTTTGTAGCTTGCCTGGAC |
| H_3_ | Cy3-GTGTGATGAGTGTGATTG/PC-Linker /TATTAGCAATCACACTCATCACAC/PC-Linker/TGAACTCGTACAGTC |
| T_3_ | GTGTGATGAGTGTGATTGCTAATACGACTCACTATAGGGGATCCCGACTGGCGAGAGCCAGGTAACGAATGGATCCTTTTTCTATCCTGAAGACA |
|  | TGTCTTCAGGATAGAAAAAGGATCCATTCGTTACCTGGCTCTCGCCAGTCGGGATCCCCTATAGTGAGTCG |


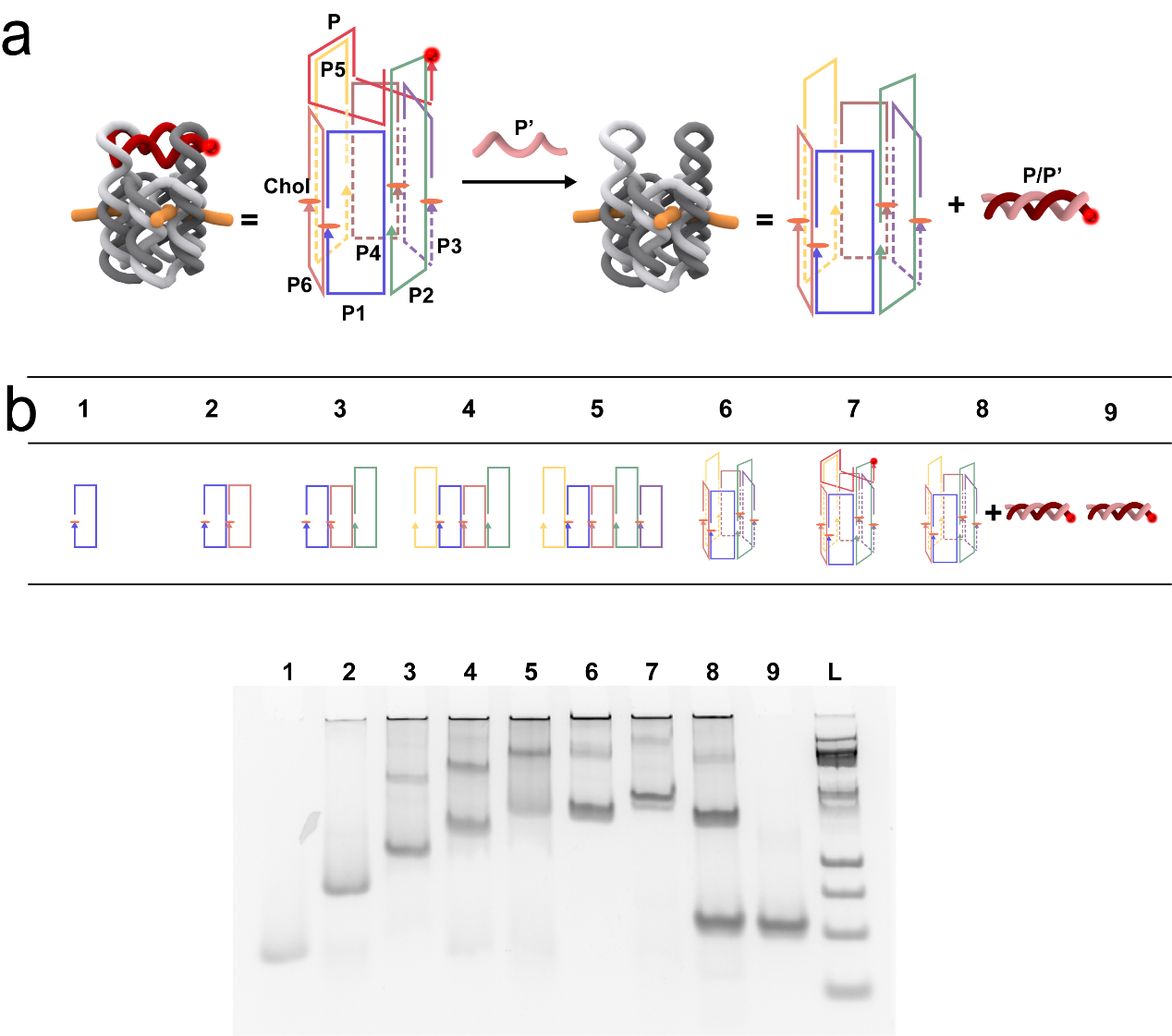


**Figure S1.** Preparation of locked/unlocked DNA origami nanopore. (a) Schematic assembly of Cy5-labeled P-locked DNA nanopore and the P’-fueled unlocking of the nanopore. (b) Gel electrophoretic image corresponding to the stepwise assembly of locked/unlocked DNA nanopore: Lane 1, P1; Lane 2, P1+P2; Lane 3: P1+P2+P3; Lane 4: P1+P2+P3+P4; Lane 5: P1+P2+P3+P4+P5; Lane 6: P1+P2+P3+P4+P5+P6; Lane 7: P1+P2+P3+P4+P5+P6+P; Lane 8: P1+P2+P3+P4+P5+P6+P+P’; Lane 9: P+P’; Lane L: 20 bp marker.

The assembly of locked DNA origami nanopore and the fuel-driven unlocking of the nanopore are depicted in Figure S1(a). Locked DNA origami nanopore includes a hexagonal prism-shaped DNA origami structure, where DNA strands P1, P2, P3, P4, P5, and P6 assemble to act as six faces of the prism, and DNA strand P hybridizes with P2 and P5 to block the hexagonal prism nanopore. The components P1, P3, P4, and P6 are modified with the cholesterol functional groups, leading to the assembled DNA hexagonal prism nanopore, where the middle part of the DNA hexagonal prism nanopore is modified with cholesterol to allow the integration of the DNA nanopore into the liposome membrane. Moreover, the strand P were modified with the Cy5 fluorophore labels to follow the locking state of the DNA nanopores in the liposome membrane. Subjecting the locked DNA nanopore to fuel strand P’ complementary to the locking strand P, results in the displaced P/P’ duplex and unlocked DNA nanopore allowing the transportation of the small molecules, e.g. Rhodamine 6G (R6G) or Mg^2+^-ions. Figure S1(b) depicts the gel electrophoretic analysis of the assembly of the DNA nanopore and the locking/unlocking processes. Lanes 1 ~ 6 depict the stepwise assembly of the DNA hexagonal prism nanopore. Lane 7 shows the locking strand P-locked DNA nanopore structure, while lane 8 demonstrates the fuel strand P’ induced unlocking of the DNA nanopore unit (c.f. lane 6) and formation of the waste duplex P/P’ (c.f. lane 9).


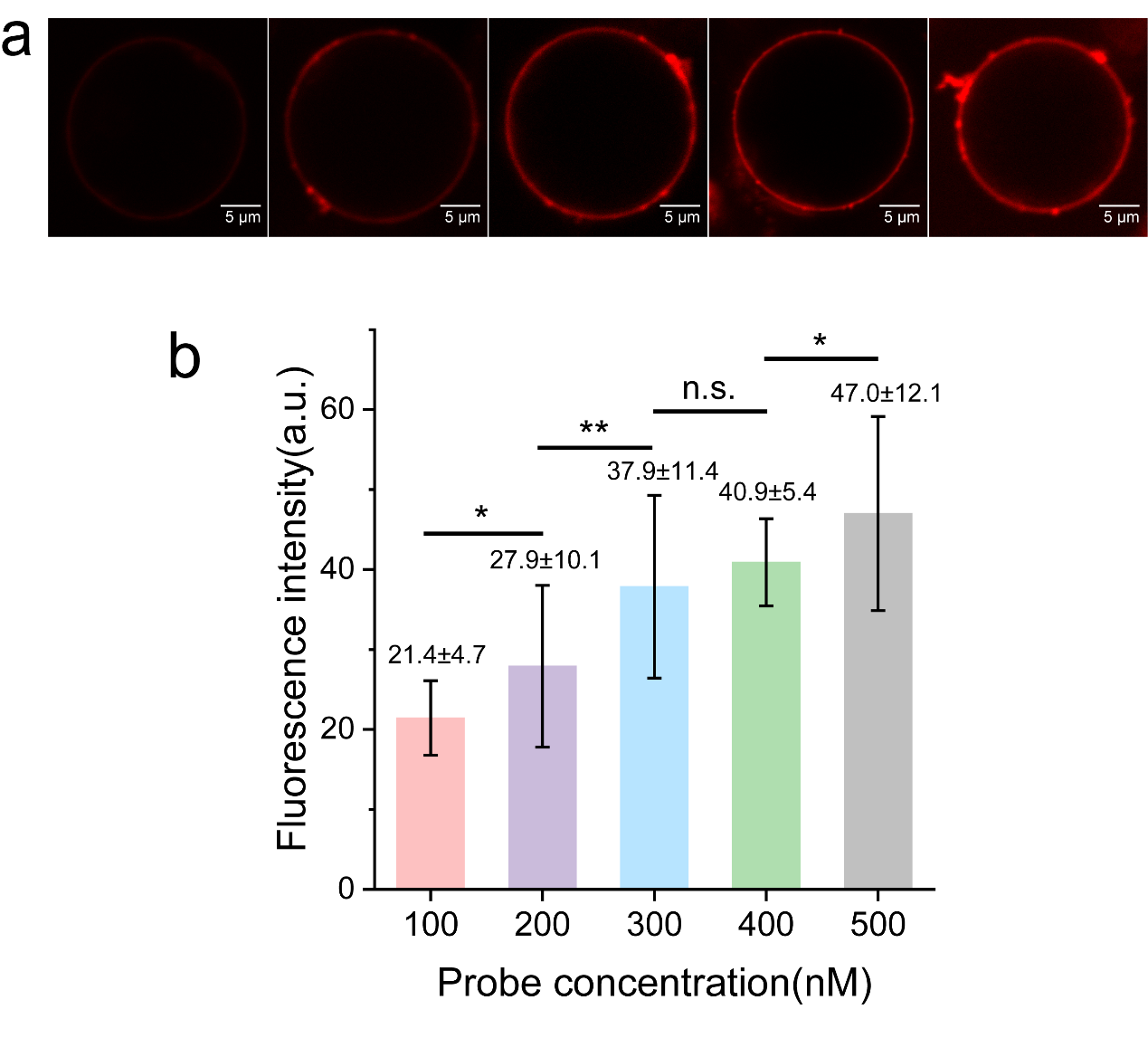


**Figure S2.** Optimization of the concentration of DNA nanopores associated with the liposome membrane. (a) Confocal fluorescence microscopy images corresponding to liposomes modified with various (100-500nM) concentrations of P-locked DNA nanopore units. (b) The integrated fluorescence intensities of Cy5-labeled P locking strand associated with the liposome nanopores.

The concentration effect of the DNA origami nanopore units associated with the liposome membrane was investigated in Figure S2. Incubation of the liposome with variable concentrations of fluorophore Cy5-labeled cholesterol-modified DNA origami nanopore leads to the liposome modified with red fluorescent DNA nanopore units, as shown in confocal fluorescence microscopy images in Figure S2(a). The respective statistical integrated fluorescence intensities were analyzed in Figure S2(b). Obviously, as the concentration of DNA nanopore units increased from 100 nM to 300 nM, the fluorescence intensities of the nanopore associated with the membrane increased rapidly, and then, the fluorescence intensities leveled off to a saturated value. Therefore, the incubation concentration of the DNA nanopore units with the liposome was chosen to be 300 nM for subsequent experiments.


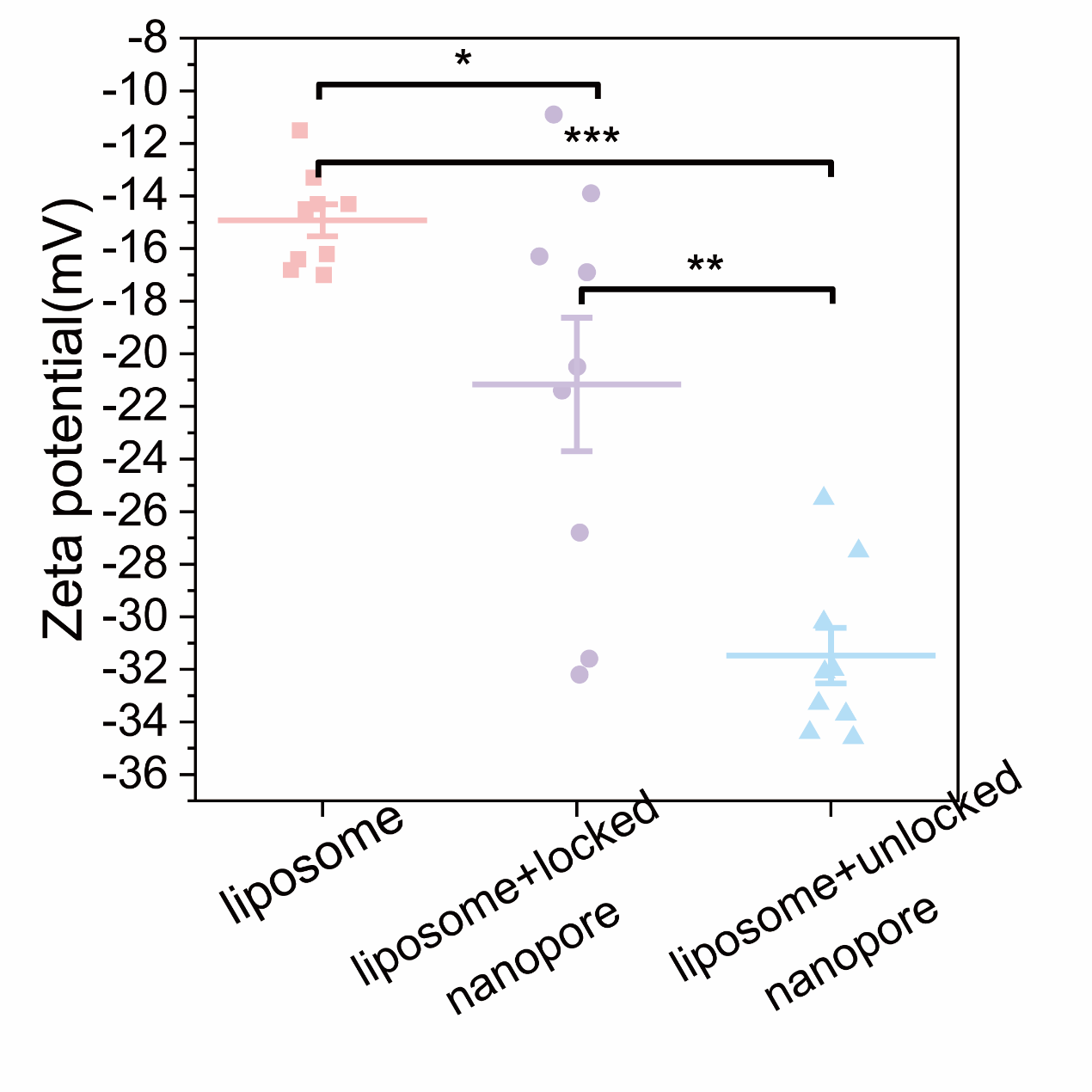


**Figure S3.** Zeta potential values of liposomes in different configurations: unmodified liposomes; P-locked nanopore-modified liposomes; P-locked nanopore-modified liposomes subjecting to unlocking strand P’.

The integration of the cholesterol-modified DNA nanopore into the liposome membrane was also demonstrated with the zeta potential analysis. Figure S3 depicts the zeta potential values of the liposome before and after modification with locked/unlocked DNA nanopore units. The pure liposomes show an average zeta potential value of -15 mV. In contrast, the liposomes modified with the locked DNA nanopore or unlocked nanopore reveal an average zeta potential value of -22 mV or -32 mV, respectively. The negative shifting of the zeta potential demonstrates the successful modification of the liposomes with the negatively-charged DNA nanopore units.


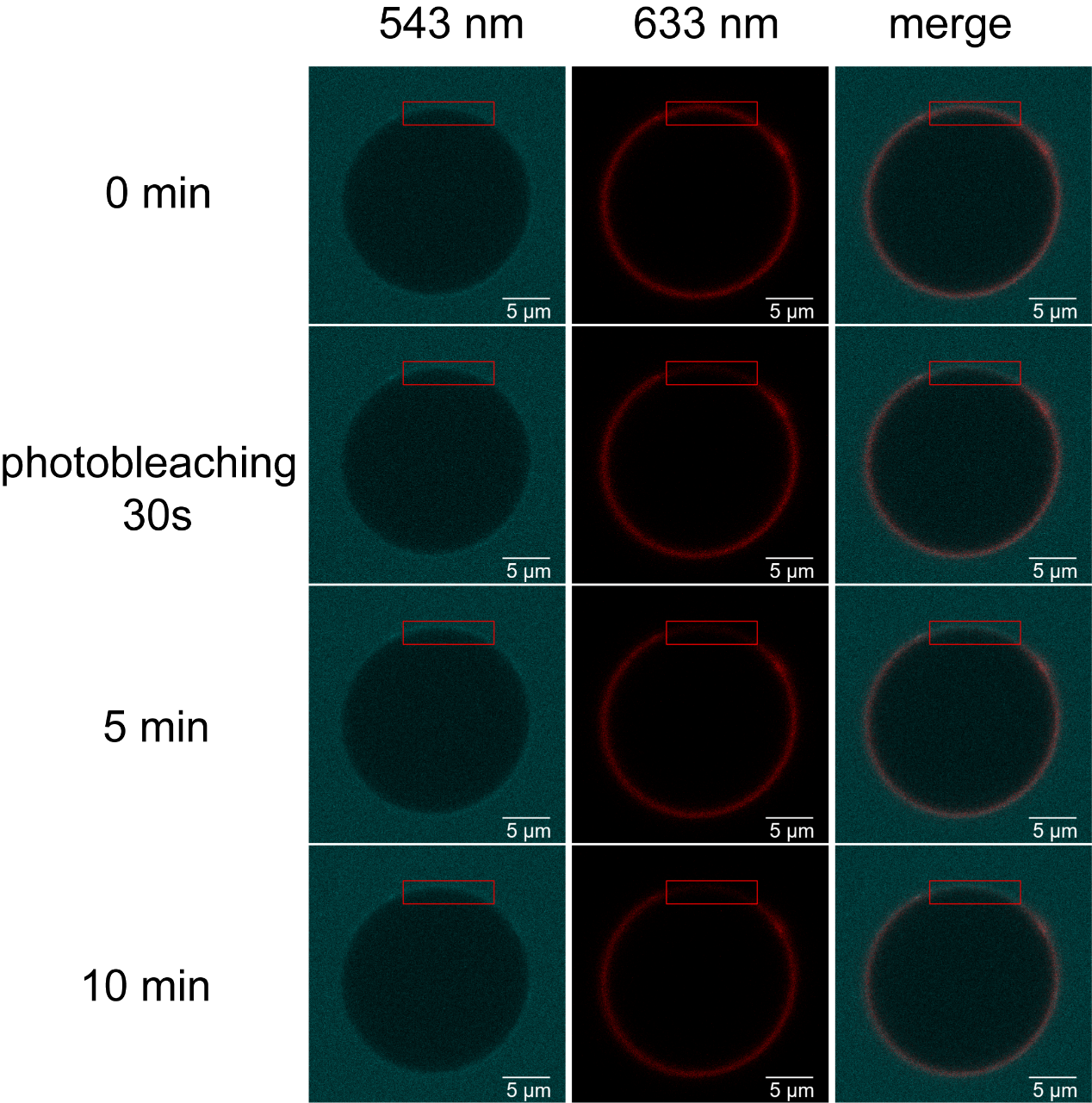


**Figure S4.** Temporal Time dependent confocal fluorescence microscopy images corresponding to Cy5-labeled P-locked DNA nanopore-modified liposome subjected to R6G. (R6G emission: 543 nm; Cy5 emission: 633 nm.) The low fluidity of DPPC/cholesterol liposome membrane at 20 °C ensures that R6G cannot permeate through the phospholipid membranes to core volume.

The small molecule, rhodamine 6G (R6G), was chosen as a fluorescent dye to probe the permeation properties of the DNA nanopore associated with the liposomes. Firstly, the permeability of the R6G to the liposome modified with locked DNA nanopore is investigated. The permeation capability of the native liposome is generally dependent on the fluidic properties of the constituents of the liposome, which could be probed by the fluorescence recovery after photobleaching experiment. As depicted by the confocal fluorescence microscopy images in Figure S4, the locked Cy5-labeled DNA nanopore modified DPPC/cholesterol liposome was subjected to the R6G solution, revealing a liposome with a red fluorescent rim, an empty core and surrounded by the blue fluorescent R6G solution. Then, the confined red rectangular region containing part of the liposome membrane was photobleached by an intensified confocal laser, followed by the time-dependent recording of the fluorescent confocal images of the liposome. Obviously, the photobleached red fluorescent rim of the liposome did not recover after 20 min, revealing low fluidity of the liposome membrane, and concomitantly, the core volume of the liposome remained empty without fluorescence. That is, the R6G could not permeate through the liposome membrane, due to the locked DNA nanopore units and low fluidity of the liposome membrane.


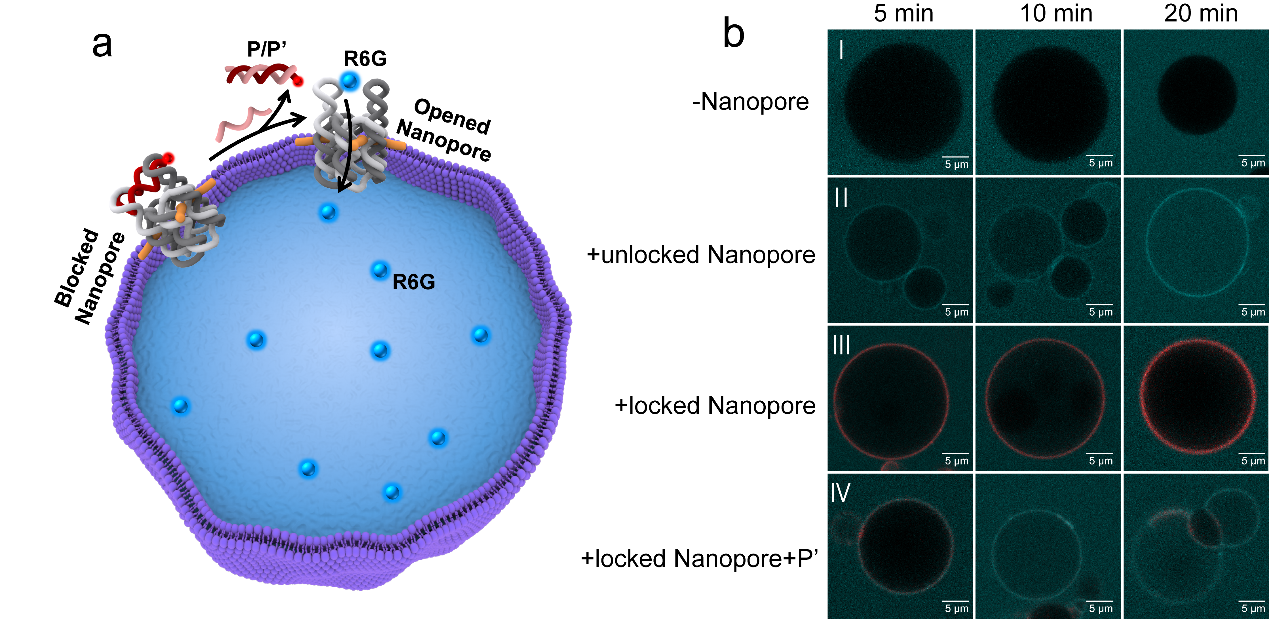


**Figure S5.** The permeation capability of R6G into liposomes by DNA nanopore channels. (a) Schematic permeation of R6G into liposomes by P’-unlocking of the P-caged DNA nanopore units associated with the liposome membrane. (b) Temporal merged (blue R6G + red Cy5) confocal fluorescence microscopy images corresponding to the liposomes of different configurations subjected to R6G: Panel I-liposomes without modification; Panel II-liposomes modified with opened DNA nanopore units; Panel III: liposomes modified with P-locked DNA nanopore units; Panel IV: liposomes modified with P-locked DNA nanopore units subjected to P’ unlocking strand.

To examine the integration of the DNA nanopore into the liposome membrane and it’s transportation behavior upon unlocking, R6G permeation experiments were conducted. Figure S5(a) depicts the schematic permeation of the fluorescent dye R6G into the liposome core volume through the unlocked DNA nanopore-modified liposomes. The liposomes were modified with the Cy5-labeled P-locked DNA nanopore units, which does not allow the permeation of the R6G. Subjecting the P-locked DNA nanopore-modified liposomes to the unlocking strand P’ that is complementary to the P locking strand, leads to the formation of P/P’ duplex and the opened state of the DNA nanopore associated with the liposome membrane, allowing the permeation of the R6G molecules into the liposome through the DNA nanopore channel. Figure S5(b) presents the fluorescence confocal microscopy images of the permeation of the R6G into the P’-unlocked P-caged DNA nanopore-modified liposomes (Panel IV), in comparison to with different control systems, liposomes without modification (Panel I), liposomes modified with the opened DNA nanopore (Panel II), and liposomes modified with the P-caged DNA nanopore (Panel III). Obviously, within 20 min, the core volume of the liposomes modified with opened DNA nanopore (Panel II and Panel IV) was occupied with the blue fluorescent R6G molecules, due to the transportation of the R6G molecules through the DNA nanopore channels. In contrast, the core volume of the liposomes without DNA nanopore (Panel I) or modified with locked DNA nanopore (Panel III) revealed blank fluorescence features, demonstrating the blocked transportation of the R6G through the P-locked DNA nanopore channels. Thus, the controlled permeability of the small molecules (e.g., R6G, or Mg^2+^-ions) into the liposome containment, could be realized by switching the unlocking/locking state of the DNA nanopore units associated with the liposome membrane.


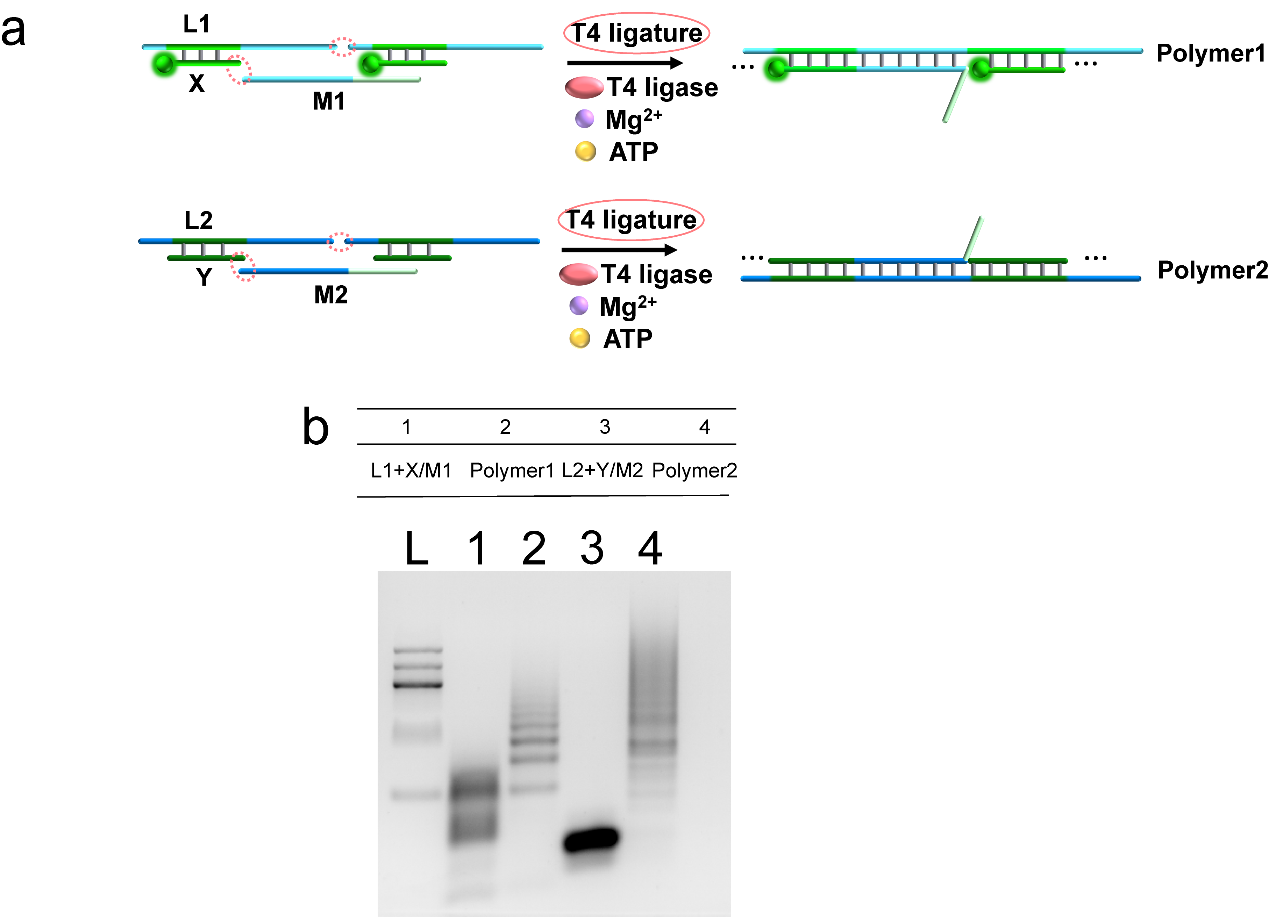


**Figure S6.** T4-DNA ligase-catalyzed ligation of the DNA module into DNA polymer. (a) Schematic ligation of the DNA module, L_1_+X/M_1_ and L_2_+Y/M_2_ into respective DNA polymers. (b) Agarose gel electrophoresis characterization of the DNA module and ligated DNA polymers: Lane L: 50 bp marker; Lane 1: L_1_+X/M_1_+T4 DNA ligase, reacted for 0 h; Lane 2: L_1_+X/M_1_+T4 DNA ligase, reacted for 2 h at 37 °C; Lane 3: L_2_+Y/M_2_+T4 DNA ligase, reacted for 0 h; Lane 4: L_2_+Y/M_2_+T4 DNA ligase, reacted for 2 h at 37 °C.

The T4 DNA ligase-catalyzed ligation of the DNA constituents into DNA polymer chains (Figure S6(a), also depicted in Figures 2(a) and (b)) were probed by the gel electrophoretic experiments, Figure S6. Lane 1 and 3 depict the DNA constituents, L_1_+X/M_2_, and L_2_+Y/M_2_, respectively, before the ligation process. And after the ligation process, evidently, the products revealed discrete high-molecular-weight bands in Lane 2 and 4, respectively, demonstrating the formation of the ligated DNA polymer chains.

**Optimization of the steps phase separated formation of the condensate O_1_ in a homogenous buffer solution.**

As a primary step studying the phase separated formation of the organelles in the liposomes, the conditions for phase separated formation of the organelle O_1_ in a homogenous buffer solution composed 30 mM Tris-HCl, 10 mM DTT, 1 mM ATP, 0.92 WU/µL T4 DNA ligase were examined. These included:


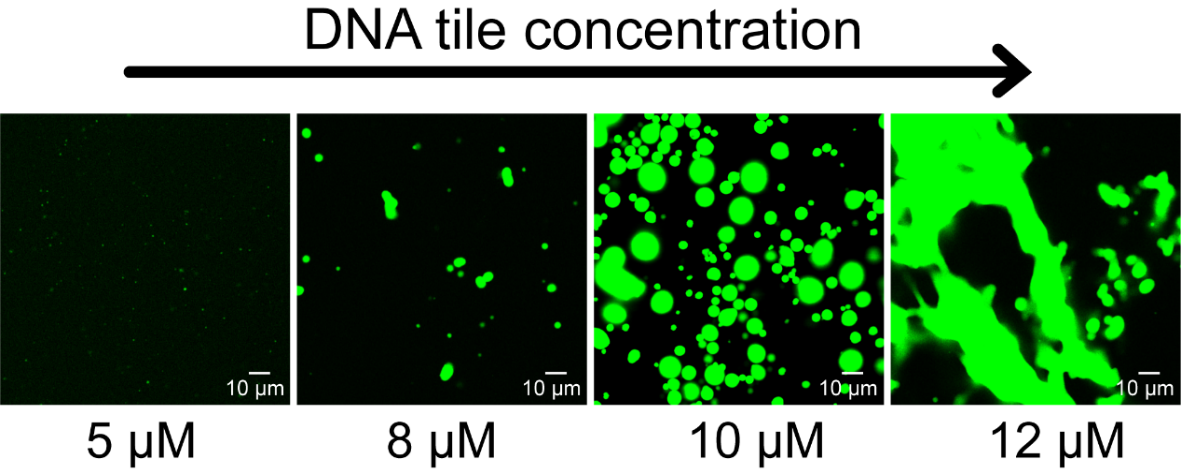


**Figure S7.** Confocal fluorescence microscopy images of phase-separated DNA microdroplet condensate O_1_ in bulk solution using different concentrations of DNA module L_1_+X/M_1_ (5 μM ~ 12 μM).

Following the shapes of the phase-separated condensates generated after a time-interval of three hours using variable concentrations of the L_1_+X/M_1_ DNA module in the presence of Mg^2+^ ions 10 mM, T4 DNA ligase 0.92 WU/µL, 30 mM Tris-HCl, 10 mM DTT, 1 mM ATP. The results are shown in Figure S7. Globular condensates ca. 10 μm are observed after three-hour time interval using 10 μM of the DNA module. At lower concentration structurally non-defined condensates are visible, whereas at higher concentrations aggregated phase-separated morphologies are observed.


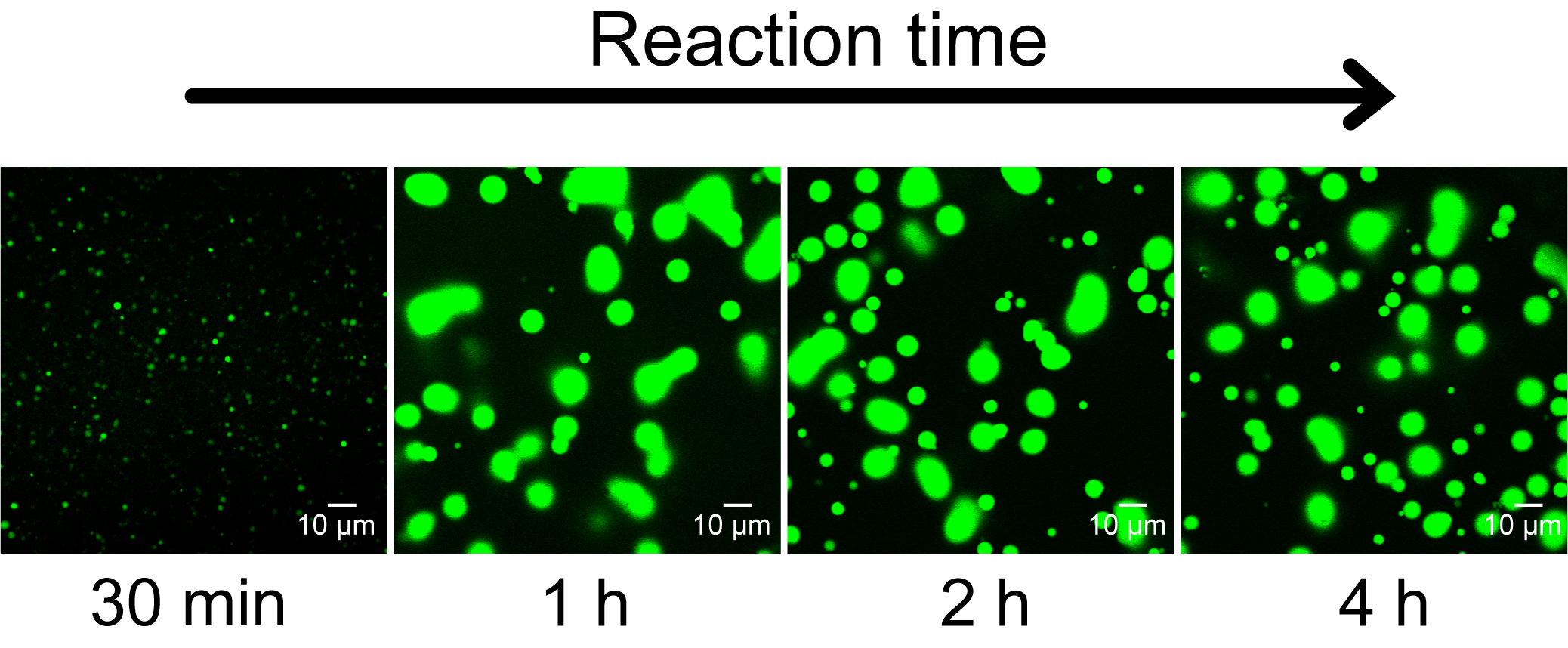


**Figure S8.** Confocal fluorescence microscopy images of phase-separated DNA microdroplet condensate O_1_ in bulk solution at different concentrations of Mg^2+^-ions (4 mM ~ 10 mM).

Following the effect of Mg^2+^-ions concentrations on the phase-separation of the reaction module L_1_+X/M_1_ 10 μM, into condensates O_1_ using T4 DNA ligase 0.92 WU/µL, 30 mM Tris-HCl, 10 mM DTT, 1 mM ATP, Figure S8. After a time interval of 3 hours, 10 μm-sized condensates were formed in the presence of Mg^2+^ ion at concentrations of 9-10 mM. At lower Mg^2+^-ion concentration structurally non-defined condensates were formed.


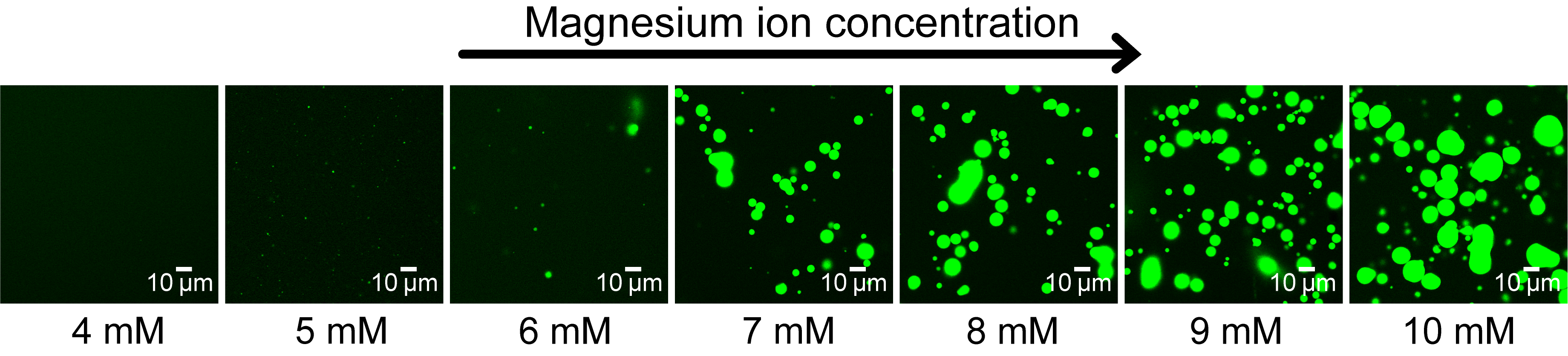


**Figure S9.** Temporal time-dependent confocal fluorescence microscopy images of phase-separated DNA microdroplet condensate O_1_ in bulk solution.

Following the temporal emergence of the condensates in the presence of L_1_+X/M_1_, 10 μM, Mg^2+^ 10 mM, T4 DNA ligase 0.92 WU/µL, 30 mM Tris-HCl, 10 mM DTT, and 1 mM ATP., Figure S9. After a time interval of ca. 3-4 hours, 10 μm-sized condensates were formed.

Accordingly, the respected conditions to prepare the liposome loaded DNA modules L_1_+X/M_1_(or L_2_+Y/M_2_) include bulk solutions containing the reaction modules at concentrations of 10 μM, Mg^2+^ 10 mM, T4 DNA ligase 0.92 WU/µL, 30 mM Tris-HCl, 10 mM DTT, and 1 mM ATP while allowing the formation of the condensates for ca. 3 hours.


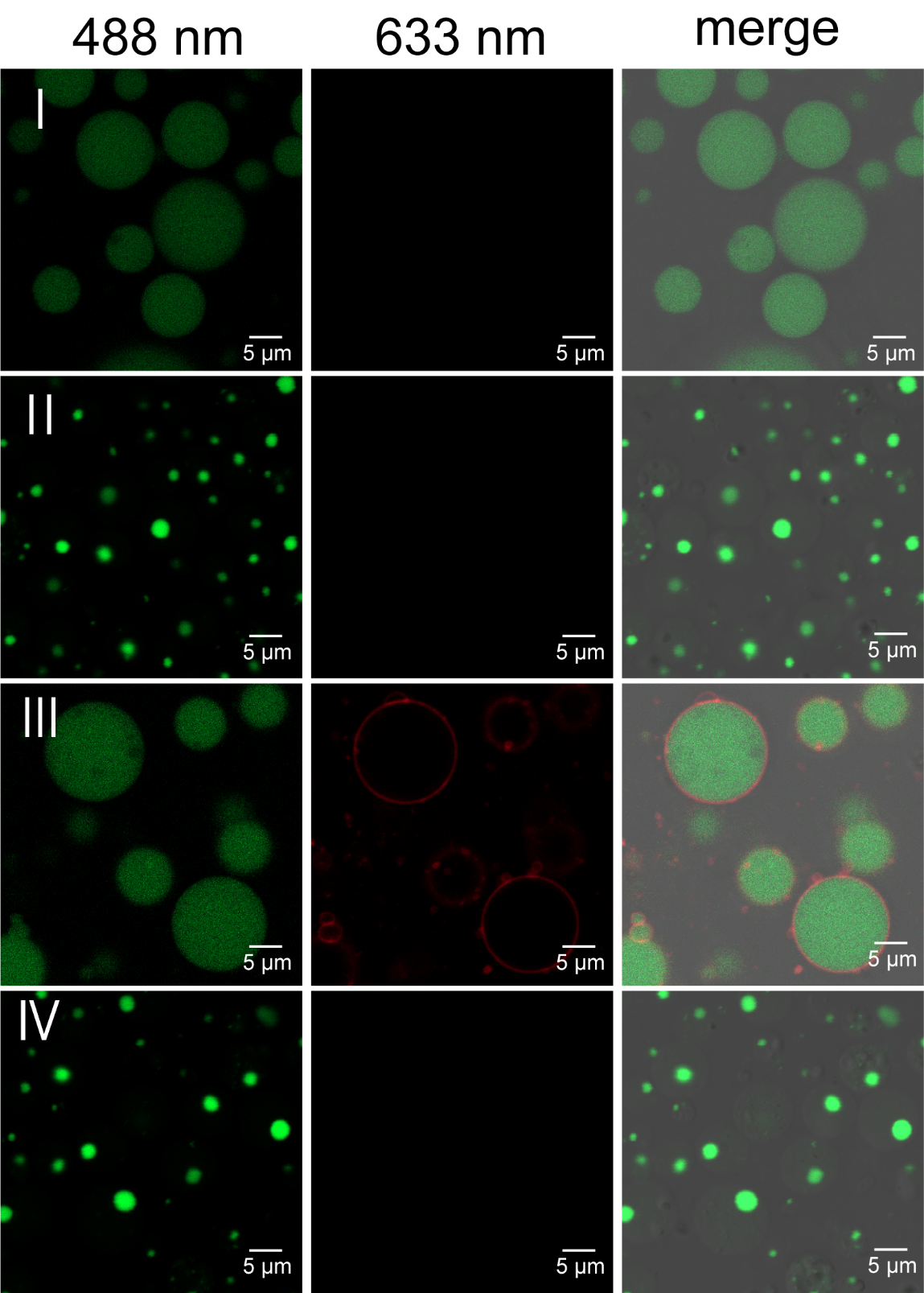


**Figure S10.** Zoom-out confocal fluorescence microscopy images of the evolved organelle O_1_ in the liposomes of different configurations: Panel I-liposomes without modification; Panel II-liposomes modified with opened DNA nanopore units; Panel III: liposomes modified with P-locked DNA nanopore units; Panel IV: liposomes modified with P-locked DNA nanopore units subjected to P’ unlocking strand.

Figure S10 depicts the zoom-out confocal fluorescence microscopy images of formation of the organelle O_1_ in the unlocked DNA nanopore-modified liposome carrier by the transportation of Mg^2+^-ions through the DNA nanopore channel, and the control systems, c.f. Figure 2(c).


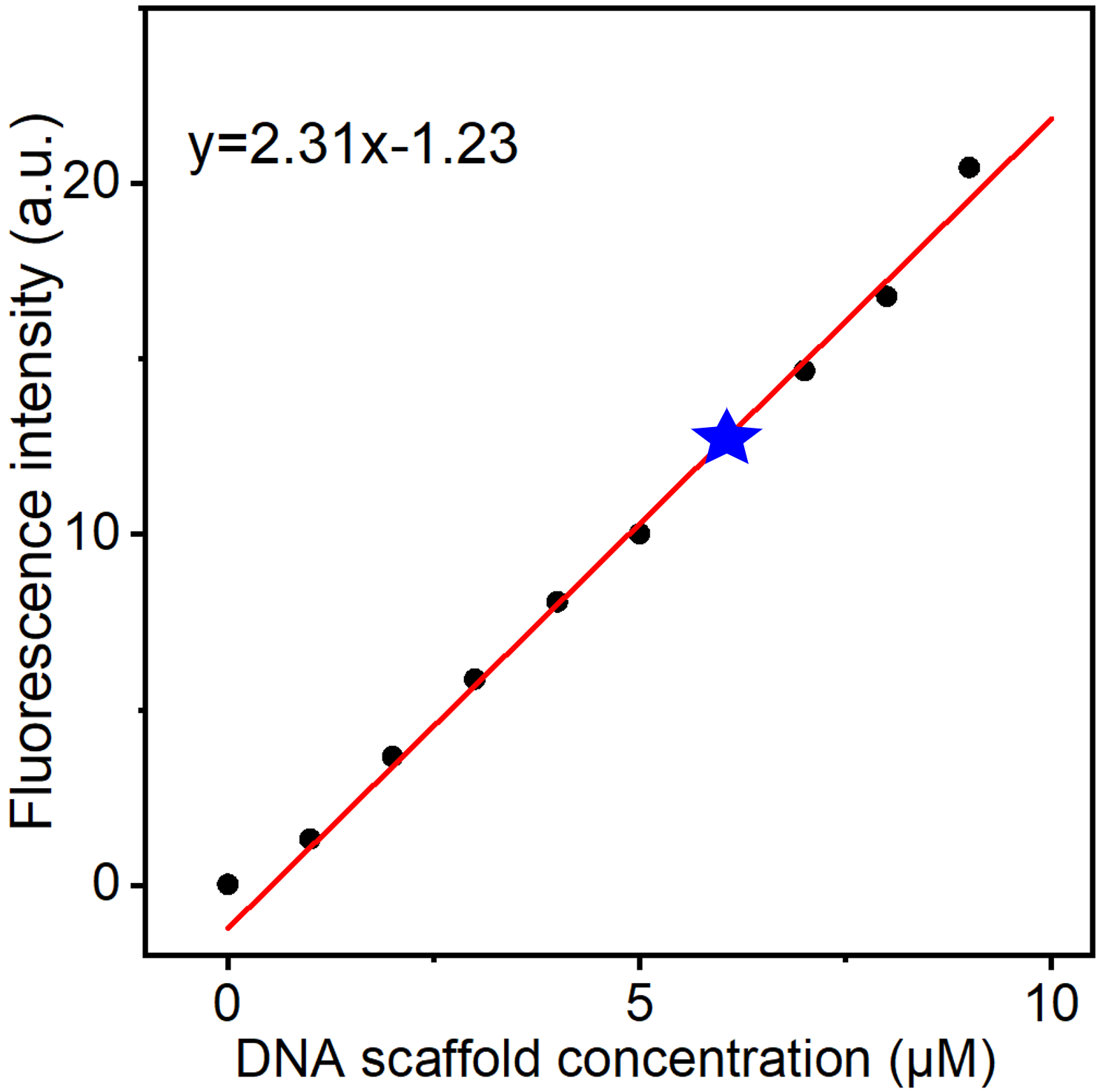


**Figure S11.** The calibration curve corresponding to the fluorescence intensity of different concentration of L_1_+X/M_1_.


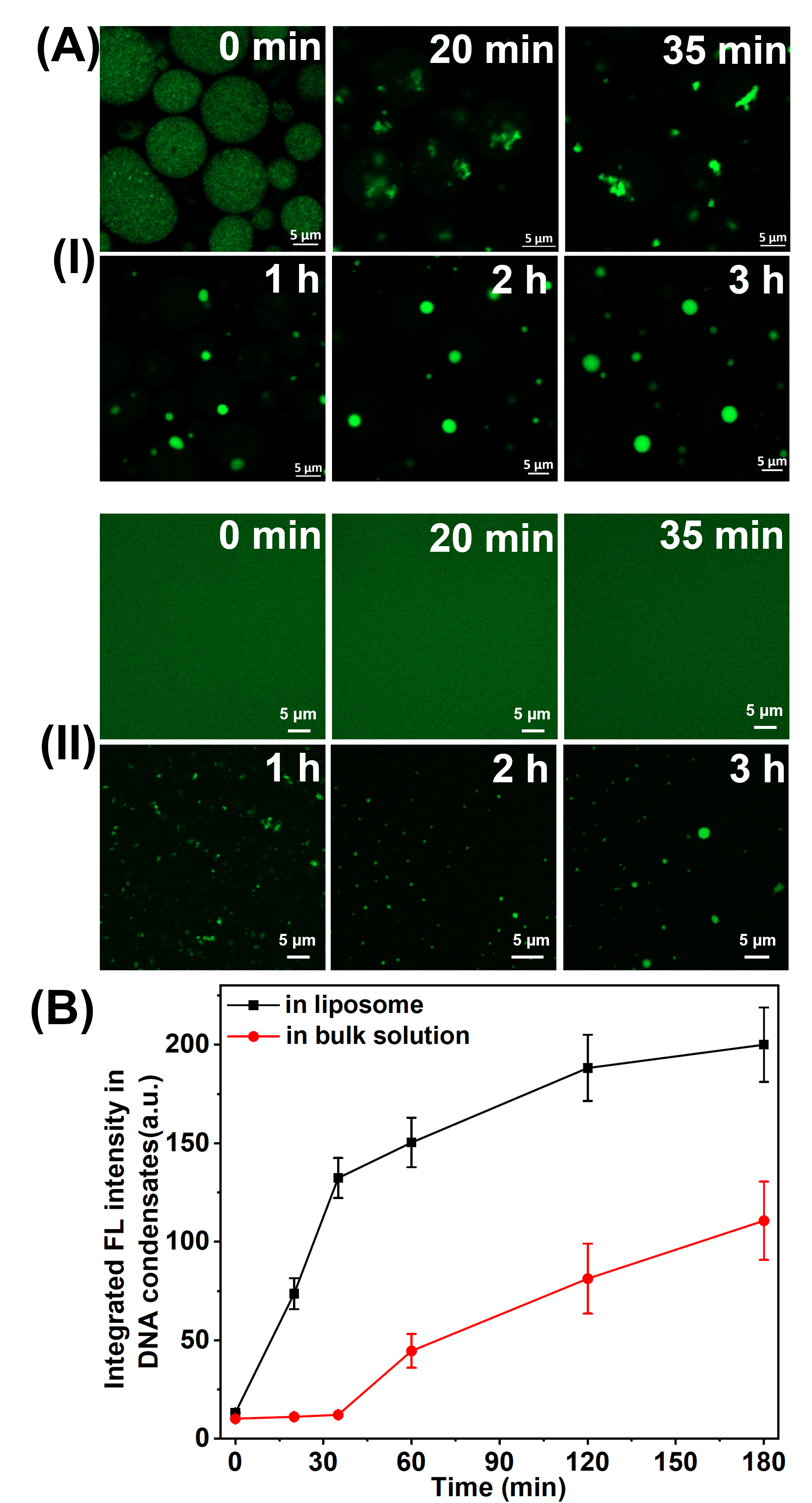


**Figure S12.** (A)The time-dependent confocal fluorescence images of O1 droplet formation in the liposome, Panel I, and in the bulk solution, Panel II. (B) The time-dependent evolution of the integrated fluorescence intensity of O1 droplet formation in the liposome, black curve, and in the bulk solution, red curve.

**Dynamics of organelles formation using different origami pore structures on the liposomes boundary.**


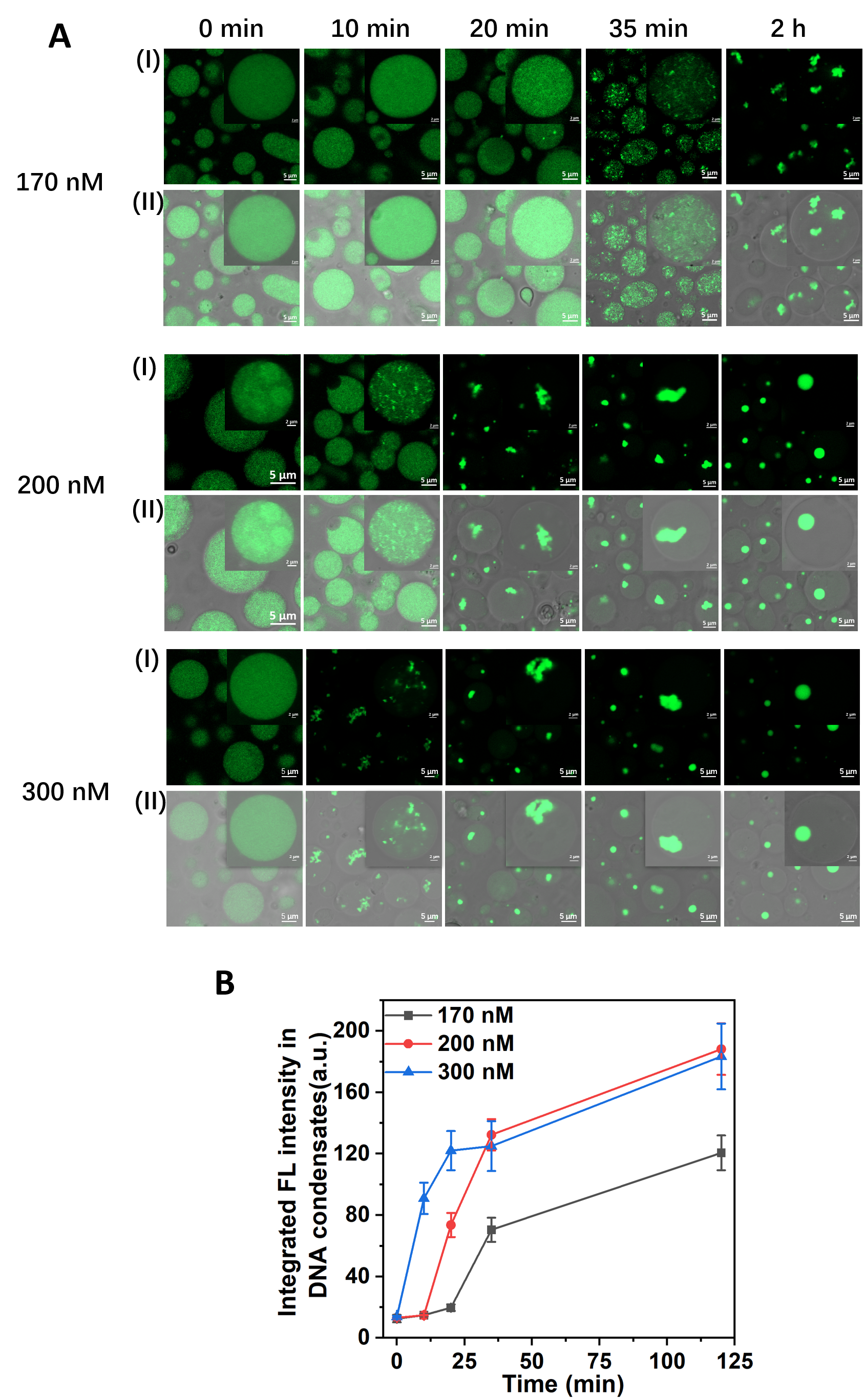


**Figure S13.** (A) Time-dependent fluorescence images of O1 droplets formation in the liposome with different concentration of DNA origami nanopore generating units. (B) The time-dependent evolution of the integrated fluorescence intensity of O1 droplet with different concentration of DNA origami nanopore generating units.

While the quantitative assessment of the average number of DNA origami nano pores associated with the liposome boundaries is difficult to evaluate, it is clear that the surface concentration of the pores should be controlled by the bulk concentration of the DNA origami nanopore generating units. Moreover, the number of boundary associated DNA origami nanopore, and their triggered unlocking should affect the dynamics of the organelle formation. Figure S13 depicts the temporal organelle O_1_ formation using different bulk concentration of the DNA origami nanopore generating agent. Evidently, as the bulk concentration of DNA origami nanopore generating agent increase, the rates of organelles formation increase, consistent with the enclosed pore generating capacity of liposome carriers.

**
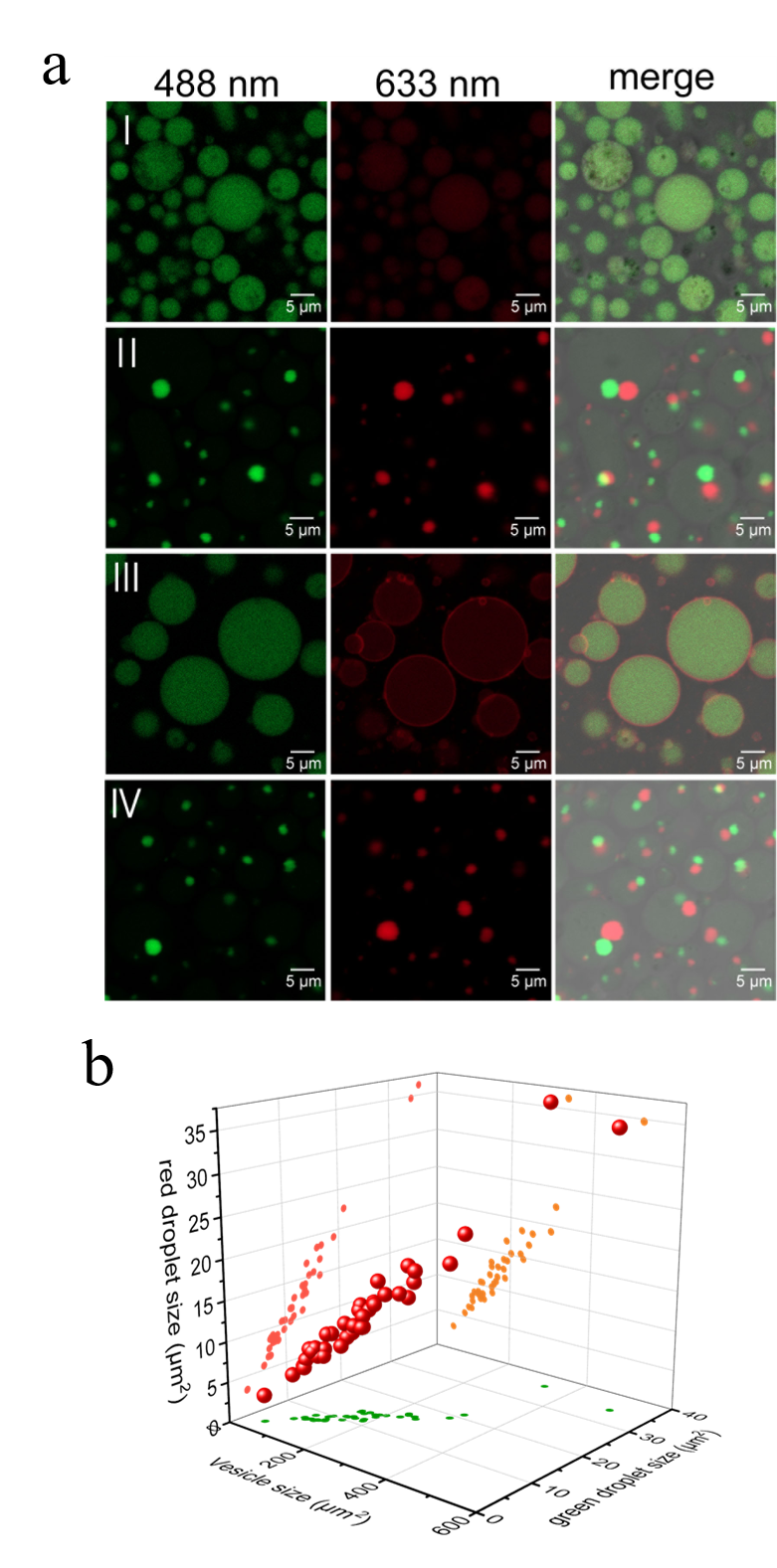
**

**Figure S14.** (A) Zoom-out confocal fluorescence microscopy images of the evolved organelle O_1_ and O_2_ in the liposomes of different configurations: Panel I-liposomes without modification; Panel II-liposomes modified with opened DNA nanopore units; Panel III: liposomes modified with P-locked DNA nanopore units; Panel IV: liposomes modified with P-locked DNA nanopore units subjected to P’ unlocking strand. (B) A three-dimension presentation correlating the relative green/red organelle dimensions in a collection of 40 different sized liposomes.

Figure S14(A) depicts the zoom-out confocal fluorescence microscopy images of orthogonal formation of two organelles, O_1_ and O_2_, in the unlocked DNA nanopore-modified liposome carrier by the transportation of Mg^2+^-ions through the DNA nanopore channel, and the control systems, c.f. Figure 3(c). It should be noted that the organelles O_1_ and O_2_ generated in the liposomes differ in their sizes and relative populations. This originates from the fact that the concentrations of the DNA constituents leading to the two types of organelles differ in the different sized liposomes, and the kinetics of assembly of the two kinds of organelles are different. To account for these sized-effects distributions of green/red organelles in a collection of 40 liposomes are present in Figure S14(B) a three-dimensional framework correlation the relative green/red organelle sizes in the collection of different-sized liposomes


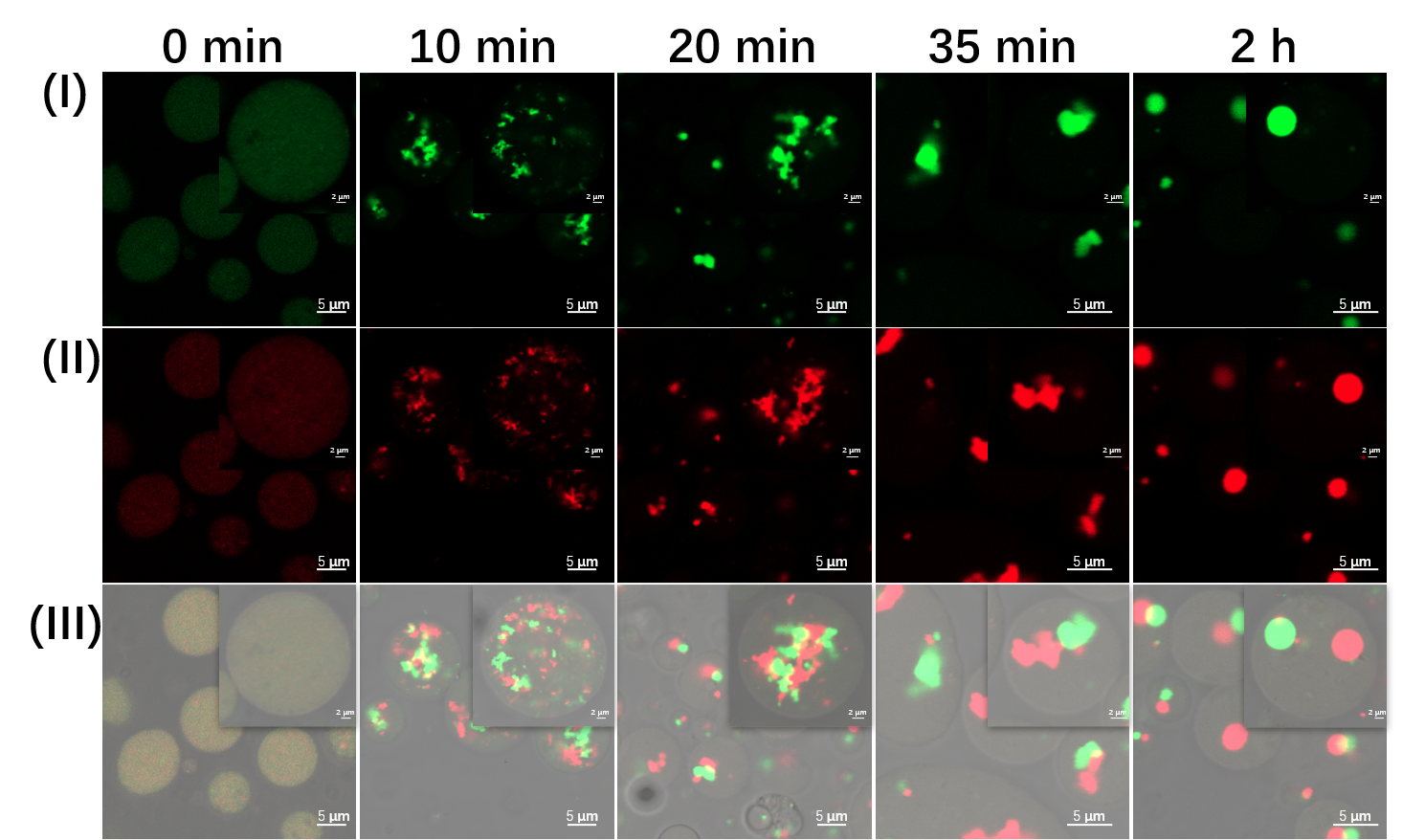


**Figure S15.** Time-dependent fluorescence images of organelles O_1_/ O_2_ formation in the liposomes. Panel I- Green channel confocal fluorescence images of organelle O_1_. Panel II- Red channel confocal fluorescence images of organelle O_2_. Panel III- Merged channel of confocal fluorescence images of organelles O_1_/ O_2_.


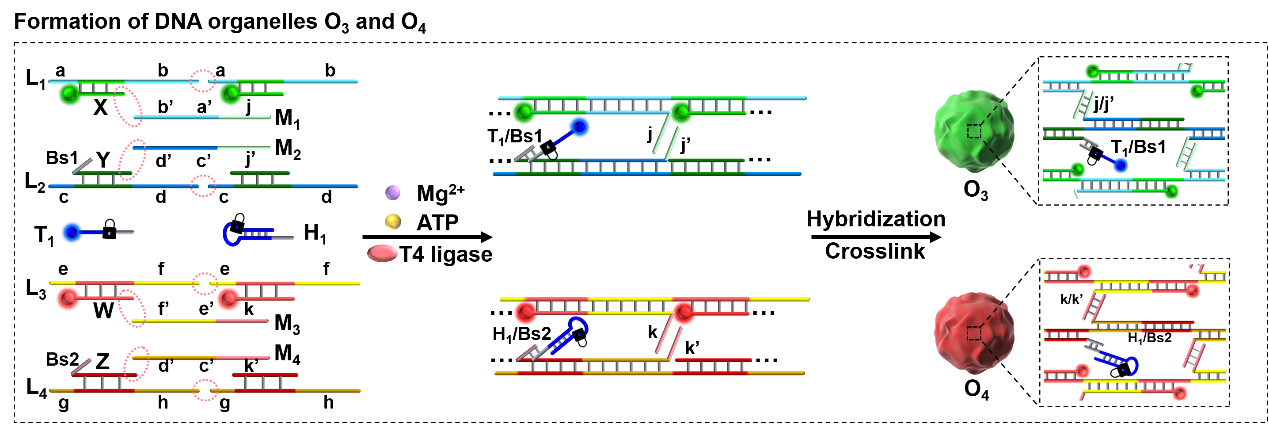


**Figure S16.** Schematic assembly process of DNA organelles O_3_ and O_4_ in liposomes.


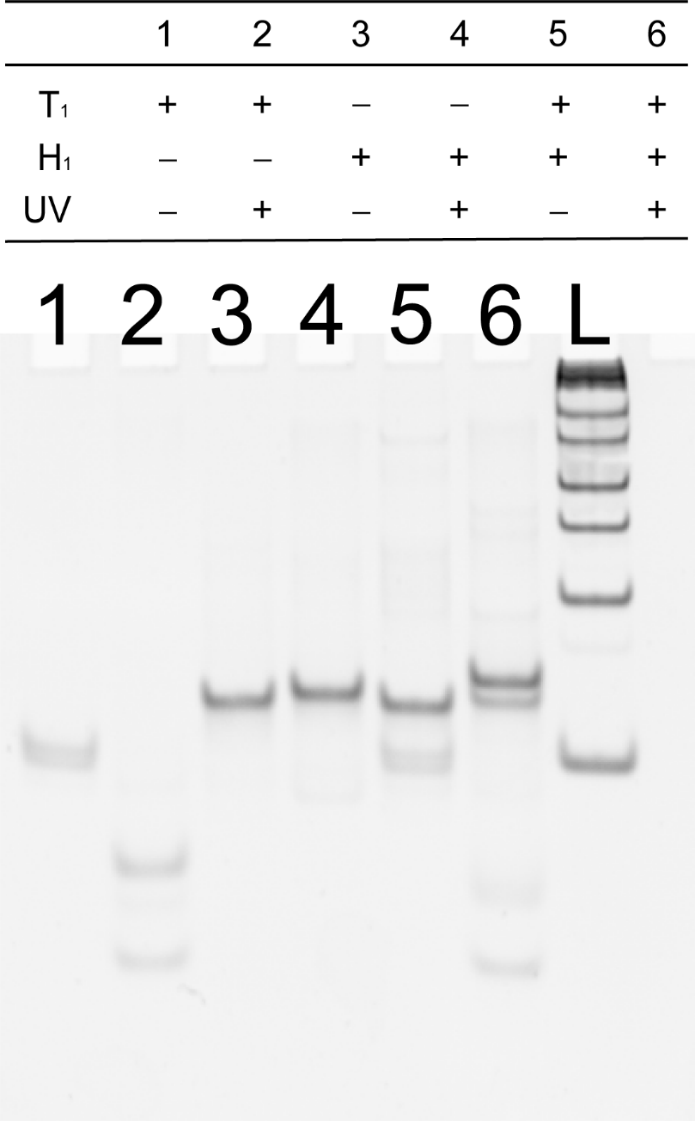


**Figure S17.** Polyacrylamide gel electrophoresis image (native) of the light-induced strand intercommunication depicted in Figure 4(a): Lane 1: T_1_; Lane 2: T_1_+UV; Lane 3: H_1_; Lane 4: H_1_+UV; Lane 5: T_1_ + H_1_; Lane 6: T_1_ + H_1_+UV; Lane L: 20 bp marker.

The light-induced strand intercommunication process between the organelles O_3_ and O_4_ was also characterized by the gel electrophoretic experiments, Figure S17. The UV-uncaging of the o-nitrobenzyl phosphate ester-modified T_1_ strand generates two band corresponding to fragmented T_1_’ and waste (lane 2 vs. lane 1). Also, the UV-uncaging of the o-nitrobenzyl phosphate ester-modified DNA hairpin H_1_ strand generated DNA duplex H_1a_/H_1b_, exhibiting similar electrophoretic rates (lane 4 vs. lane 3). Incubation of the T_1_ strand with hairpin H_1_ in the dark generates the inert two separated band of T_1_ and H_1_ (see lane 5), respectively. Subjecting the mixture of the T_1_ strand and hairpin H_1_ to UV irradiation leads, however, to the formation of uncaged T_1_’ and DNA duplex H_1a_/H_1b_, followed by the toehold-mediated strand displacement formation of T_1_’/ H_1a_ that exhibits slower migrating band, and respective wastes bands (see lane 6). The result is consistent with the mechanism introduced in Figure 4(a).


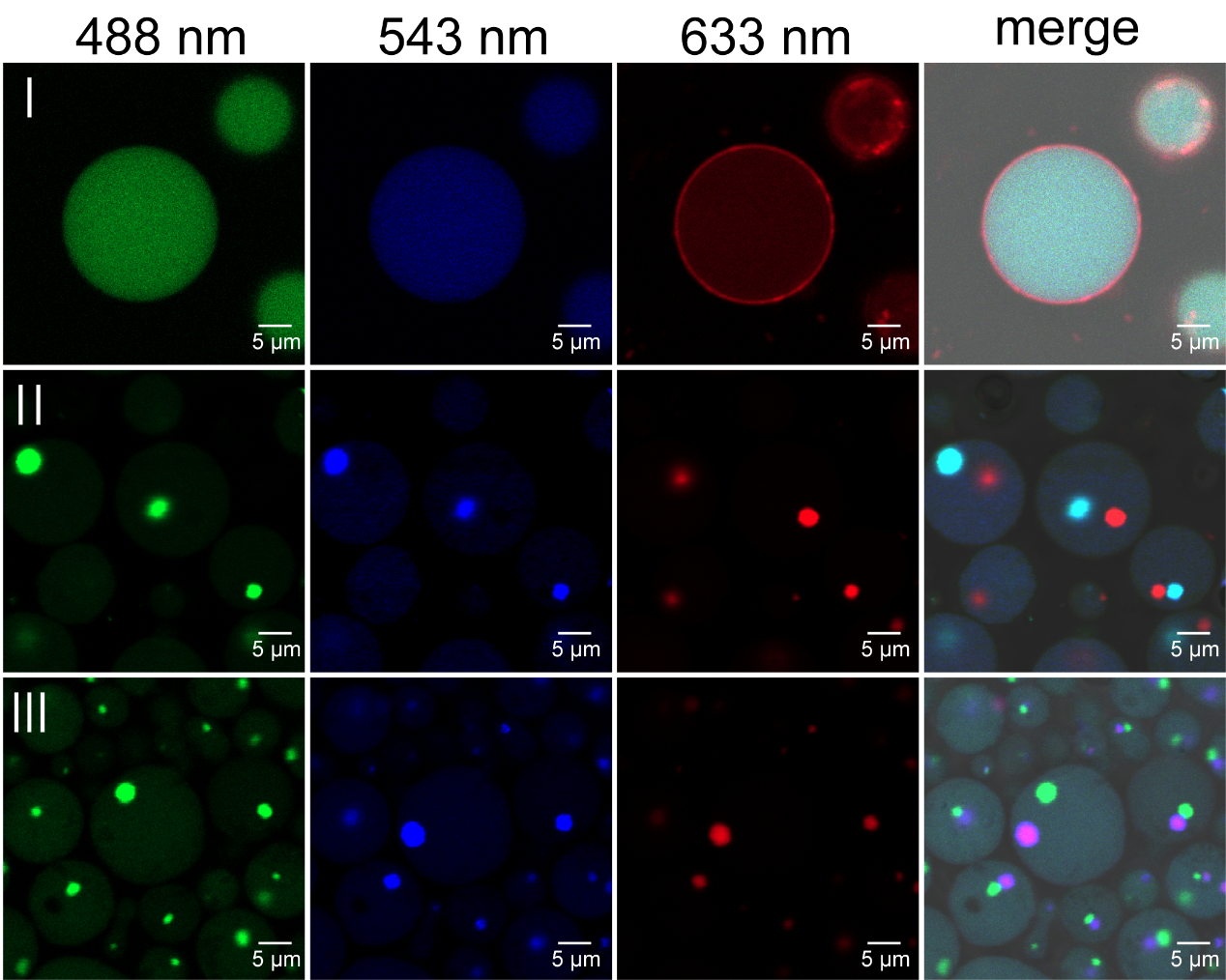


**Figure S18.** Zoom-out confocal fluorescence microscopy images probing the phase separation of the two organelles O_3_/O_4_ in the liposome containment and their light triggered reconfiguration into the O_3_’/O_4_’ organelles. Panel I-The constituent-loaded liposome prior to the triggered opening of the pores and the Mg^2+^-ion evolution of organelles O_3_/O_4_. Panel II-After opening the pores in the liposome boundary and the Mg^2+^-ions activation of the ligation of the constituents and self-assembly of phase-separated O_3_/O_4_ organelles. Panel III-After UV-light triggered activation of organelles O_3_/O_4_ and their intercommunication reconfiguration into organelles O_3_’/O_4_’. FAM: 488 nm, Green; Cy3: 543 nm, Blue; Cy5: 633 nm, red.

Figure S18 depicts the zoom-out confocal fluorescence microscopy images of light-induced strand intercommunication and dictated reconfiguration of two organelles, O_3_ and O_4_, in the liposomes with different configurations, c.f. Figure 4(b).


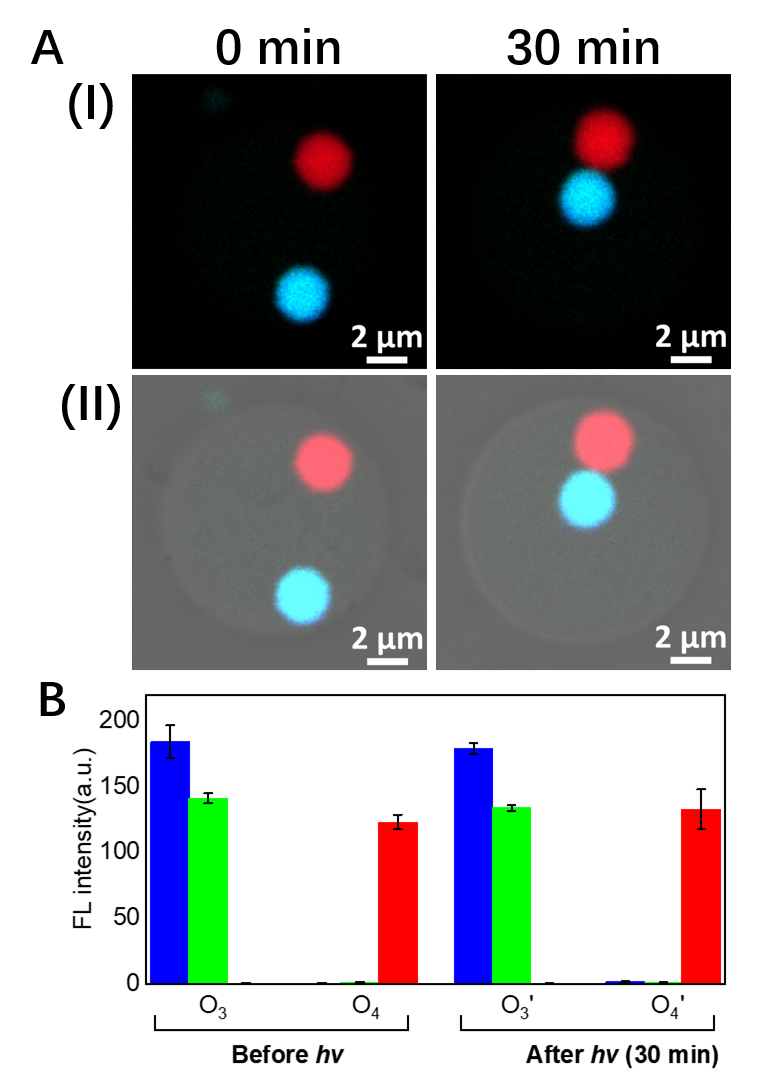


**Figure S19.** (A) Confocal fluorescence microscopy images probing non-intercommunication process between the two control organelles O_3_/O_4_ lacking the photoresponsive protecting ortho-nitrobenzyl phosphate ester unit, before and after UV illumination (λ = 365 nm, 30 min). (B) Fluorescence intensities of the DNA constituents associated with the control O_3_/O_4_ coacervates before and after UV illumination. Error bars are deduced from N = 4 independent experiments.

Fluorescence features of O_3_/O_4_, loaded with the DNA constituents shown in Figure 4, lacking the photoresponsive protecting ortho-nitrobenzyl phosphate ester unit, before and after UV illumination (λ = 365 nm) for 30 minutes.

Results demonstrated that UV illumination of control coacervates has no effect on their structural integrity.


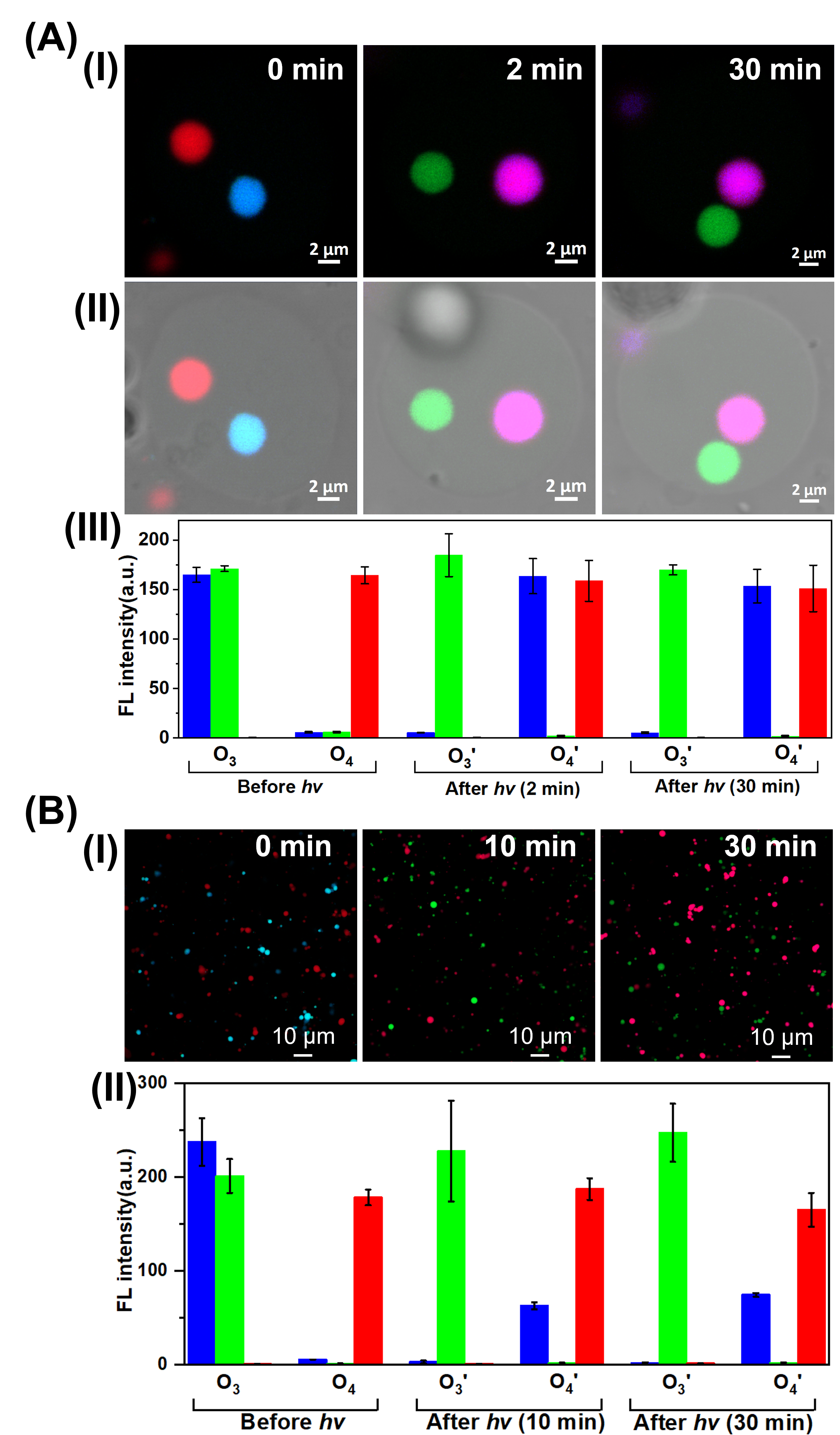


**Figure S20.** (A) Confocal fluorescence microscopy images of organelles O3/O4 inside liposomes before and after UV illumination at different time intervals. Panel I-Fluorescence images of organelles O_3_/O_4_ recorded without bright-field channel. Panel II-Merged fluorescence and bright-field images of organelles O_3_/O_4_. Panel III-Fluorescence intensities of the constituents associated with organelles O3/O4 prior to UV illumination and of O_3_’/O_4_’ after reconfiguration inside liposomes. (B) Confocal fluorescence microscopy images of organelles O_3_/O_4_ in bulk solution before and after UV illumination at different time intervals. Panel I-Fluorescence images of organelles O3/O4 recorded without bright-field channel. Panel II-Merged fluorescence and bright-field images of organelles O3/O4. Panel III-Fluorescence intensities of the constituents associated with organelles O3/O4 prior to UV illumination and of O3’/O4’ after reconfiguration in bulk solution. Error bars are deduced from N = 4 independent experiments.


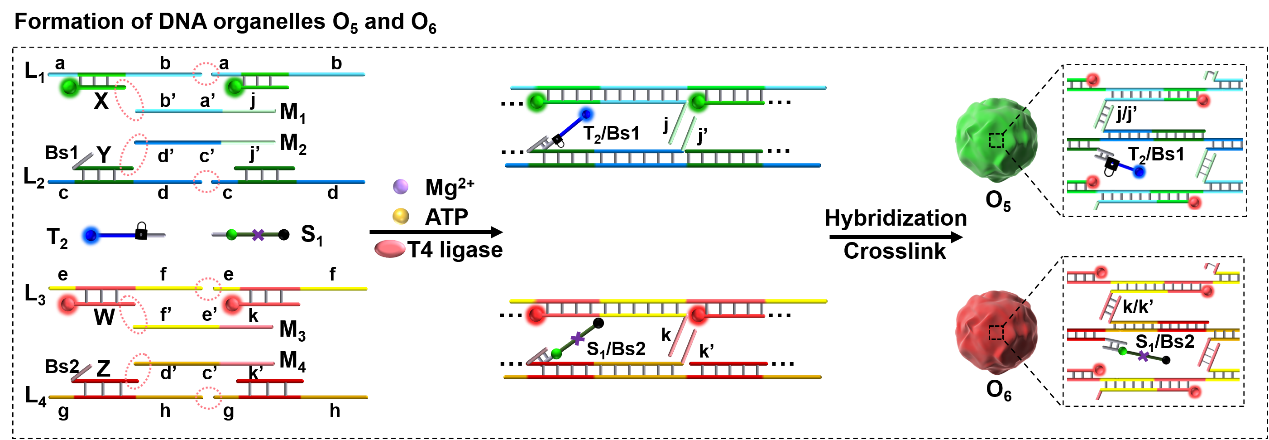


**Figure S21.** Schematic assembly process of DNA organelles O_5_ and O_6_ in liposomes.


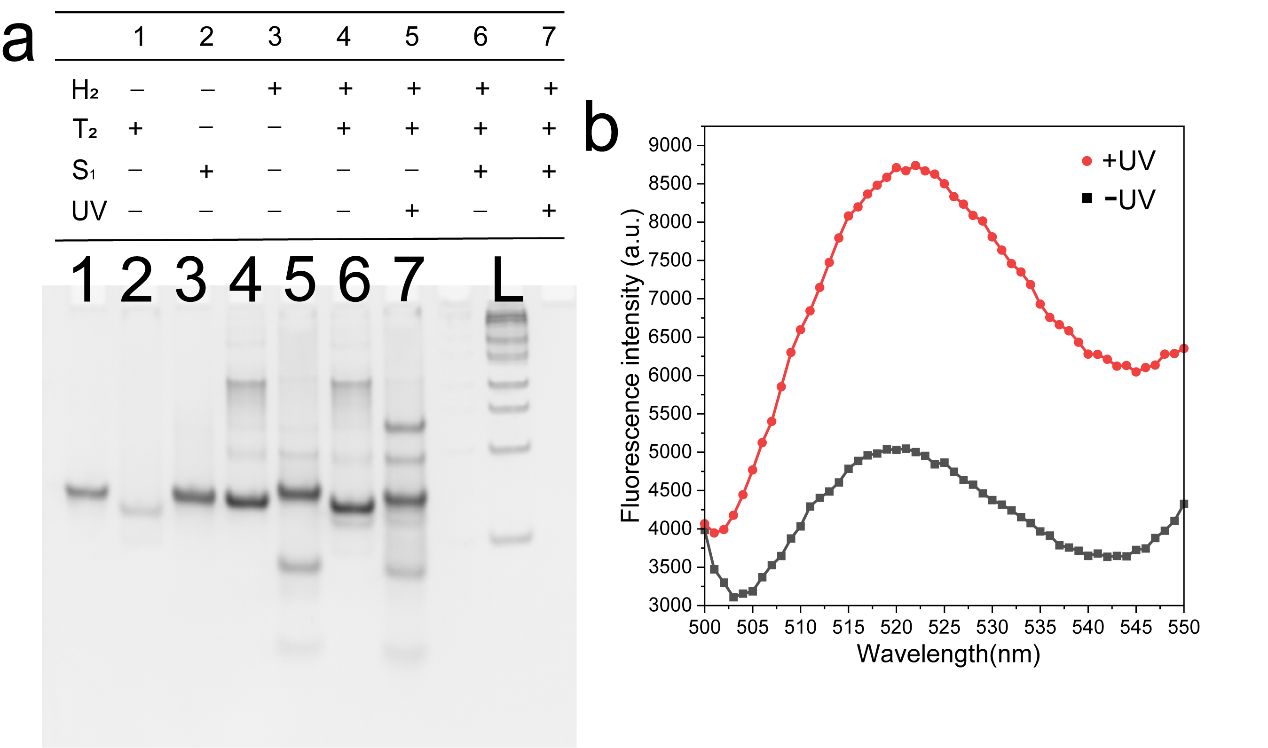


**Figure S22.** (a) Polyacrylamide gel electrophoresis image (native) corresponding to the light-triggered evolution of DNAzyme catalytic units depicted in Figure 5(b): Lane 1: T_2_; Lane 2: S_1_; Lane 3: H_2_; Lane 4: H_2_+T_2_; Lane 5: H_2_+T_2_+UV; Lane 6: H_2_+T_2_+S_1_; Lane 7: H_2_+T_2_+S_1_+UV; Lane L: 20 bp marker. (b) Fluorescence spectra of the substrate S_1_ in the mixture of H_2_, T_2_ and S_1_ (each 50nM), incubated at 37 °C for 1 h, in the absence of UV (black curve) or with the UV irradiation for 5 min.

The light-induced, DNAzyme-mediated, intercommunication and reconfiguration between the organelles O_5_ and O_6_ was also characterized by the gel electrophoretic experiment, Figure S22(A), and DNAzyme-cleaved fluorescent substrate experiments, Figure S22(B). As shown in Figure S22(A), Incubation of the T_2_ strand with hairpin H_2_ in the dark generates the inert overlapped band of T_2_ and H_2_ (see lane 4 vs. lane 1 and lane 3). Subjecting the mixture of T_2_/H_2_ to UV irradiation leads, however, to the formation of uncaged T_2_’ and DNA duplex H_2a_/H_2b_, followed by the toehold-mediated strand displacement formation of T_2_’/H_2a_ that exhibits slower migrating band, H_2b_ that exhibits faster migrating band, and respective waste band (see lane 5). Furthermore, subjecting the mixture of T_2_/H_2_/S_1_ to UV irradiation leads to the formation of T_2_’/H_2a_, and H_2b_ strand that acts as DNAzyme units recognizing and cleaving substrate S_1_ to generate waste bands (see lane 7 vs. lane 6 and lane 2).

Figure S22(B) depicts the fluorescence spectra of the FAM/BHQ-1 pair-modified substrate S_1_ in the mixture of T_2_/H_2_/S_1_ before and after UV irradiation. Evidently, upon UV activation, released DNAzyme unit H_2b_ catalyzes the cleavage of the substrate S_1_, generating increased FAM fluorescence intensity (red curve). In contrast, without UV activation, the substrate S_1_ remain intact, revealing quenched low fluorescence intensity of FAM (black curve).

These results are consistent with the mechanism introduced in Figure 5(a) and (b).


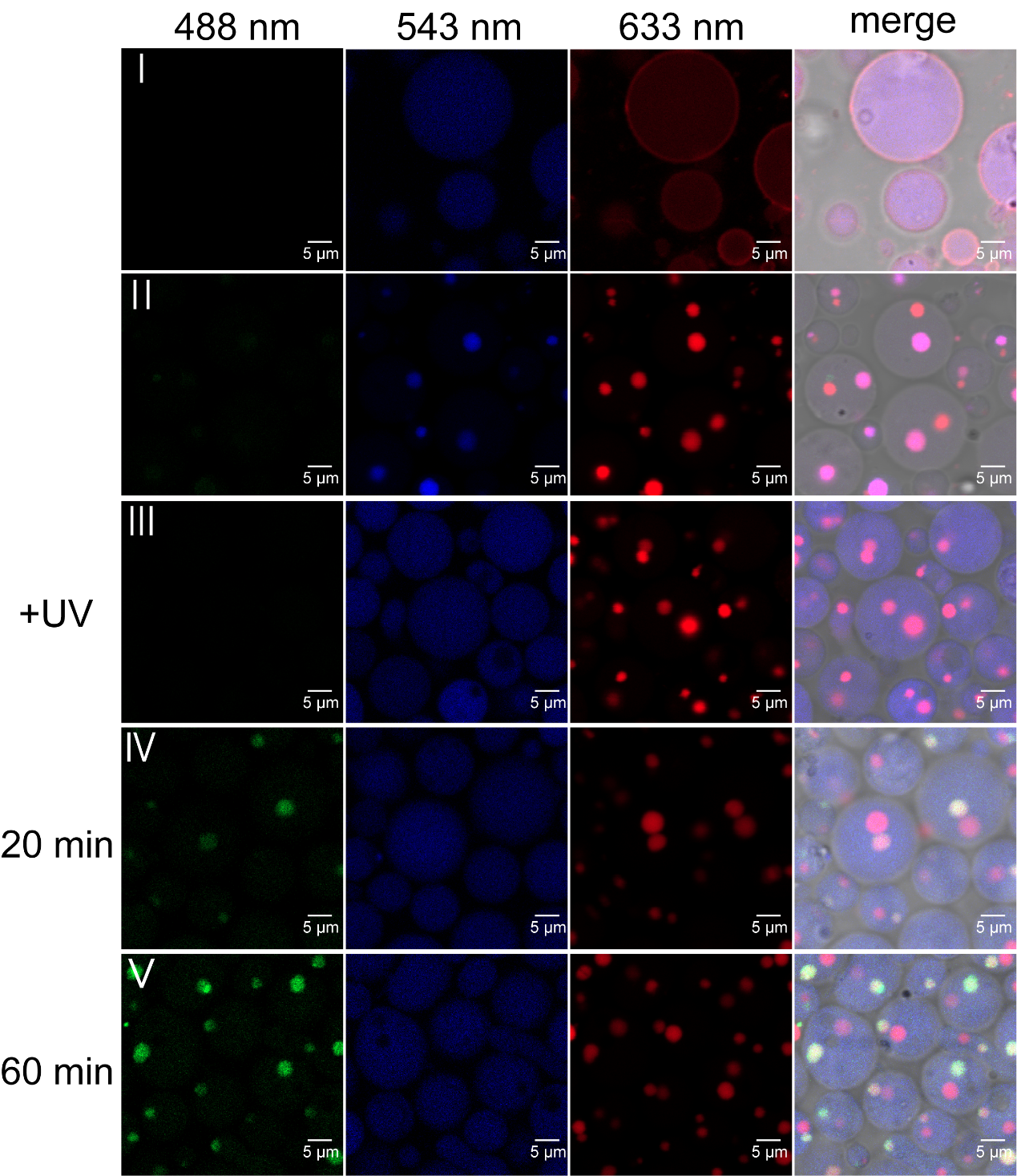


**Figure S23.** Zoom-out confocal fluorescence microscopy images corresponding to: Panel I-the constituents in the pore-locked liposomes, prior to phase separation; Panel II-after unlocking the pores and inducing the phase-separated organelles O_5_/O_6_; Panel III-Panel V-after light-induced activation of organelles O_5_/O_6_ and recording at time-intervals the fluorescence features of the liposome, upon dynamic reconfiguration into the O_5_’/O_6_’ state (Panel III-after 0 min, Panel IV-after 20 min, Panel V-after 60 min). FAM: 488 nm, Green; Cy3: 543 nm, Blue; Cy5: 633 nm, red.

Figure S23 depicts the zoom-out confocal fluorescence microscopy images of light-induced DNAzyme-mediated, intercommunication and reconfiguration between the organelles O_5_ and O_6_ in the liposomes of different configurations, c.f. Figure 5(c).


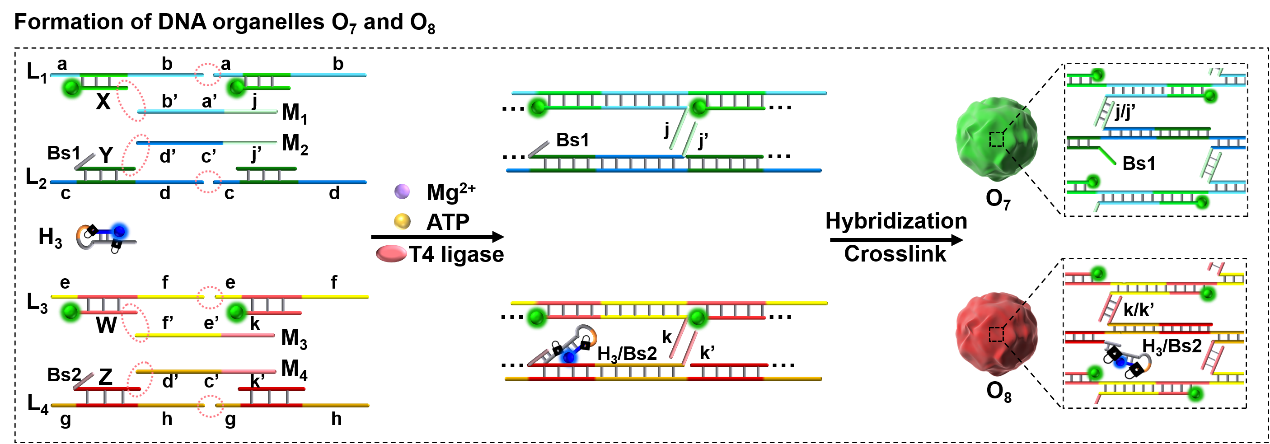


**Figure S24.** Schematic assembly process of DNA organelles O_7_ and O_8_ in liposomes.


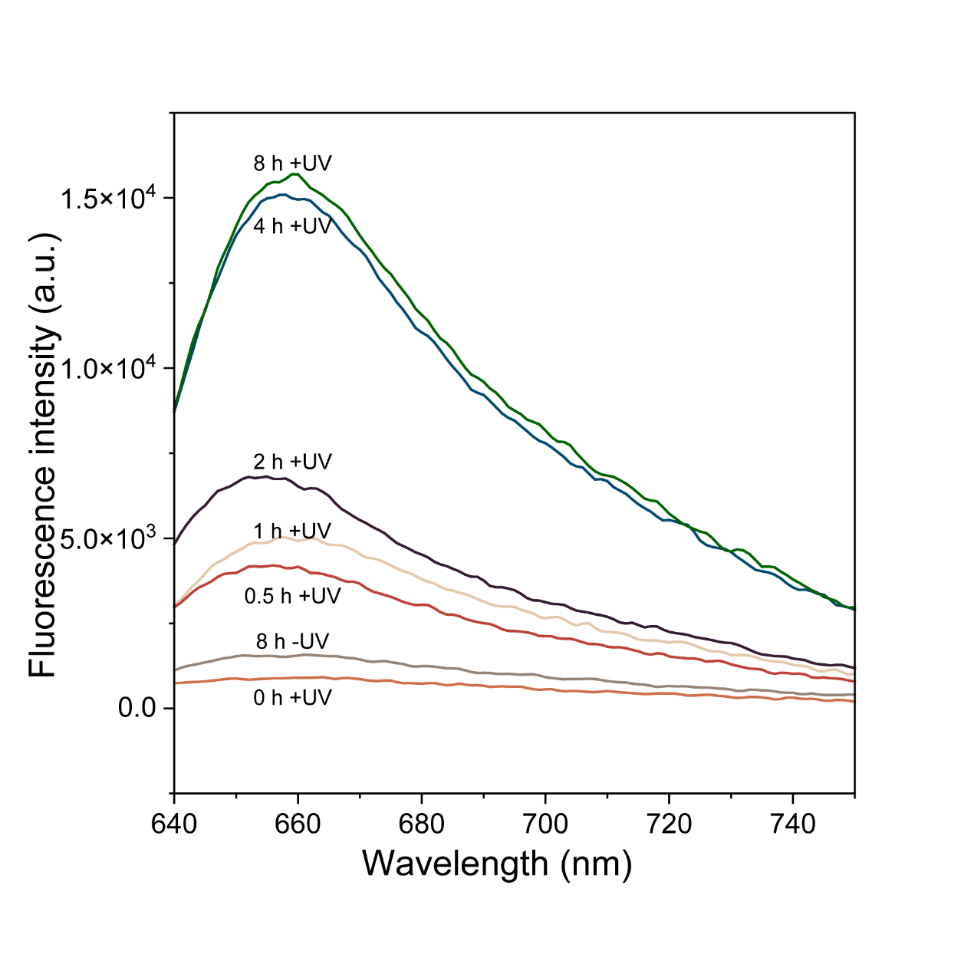


**Figure S25.** Temporal fluorescence spectra of MG/aptamer generated from the light-induced active transcription machinery and the control system without UV activation. T_3_ is rapidly annealed in 1×PBS to form incomplete dsDNA transcription template. 200nM T_3_, 500 nM H_3_, 500 nM NTP, 1 U/µL T7 RNA Polymerase was irradiated with UV for 5 minutes, and then incubated at 37 °C for transcription process.

Figure S25 depicts the temporal fluorescence spectra of malachite green (MG)/RNA aptamer complex, where the MG RNA aptamer is generated by the light-induced reconfiguration and formation of active transcription machinery from the mixture of T_3_/H_3_, T7 RNA polymerase (T7 RNAp), NTPs. Evidently, upon UV light irradiation, the fluorescence intensity of MG/RNA aptamer increases significantly from t = 0 to t = 4 h, and then levels off to a constant value from t = 4 h to t = 8 h, originating from the light-induced formation of complete H_3a_/T_3_ primer/transcription template module and active transcription machinery. In contrast, without UV irradiation, the mixture reveals a negligible background fluorescence intensity of MG/RNA aptamer after 8 h incubation, due to the incomplete transcription machinery.

These results are consistent with the mechanism introduced in Figure 6(a) and (b).


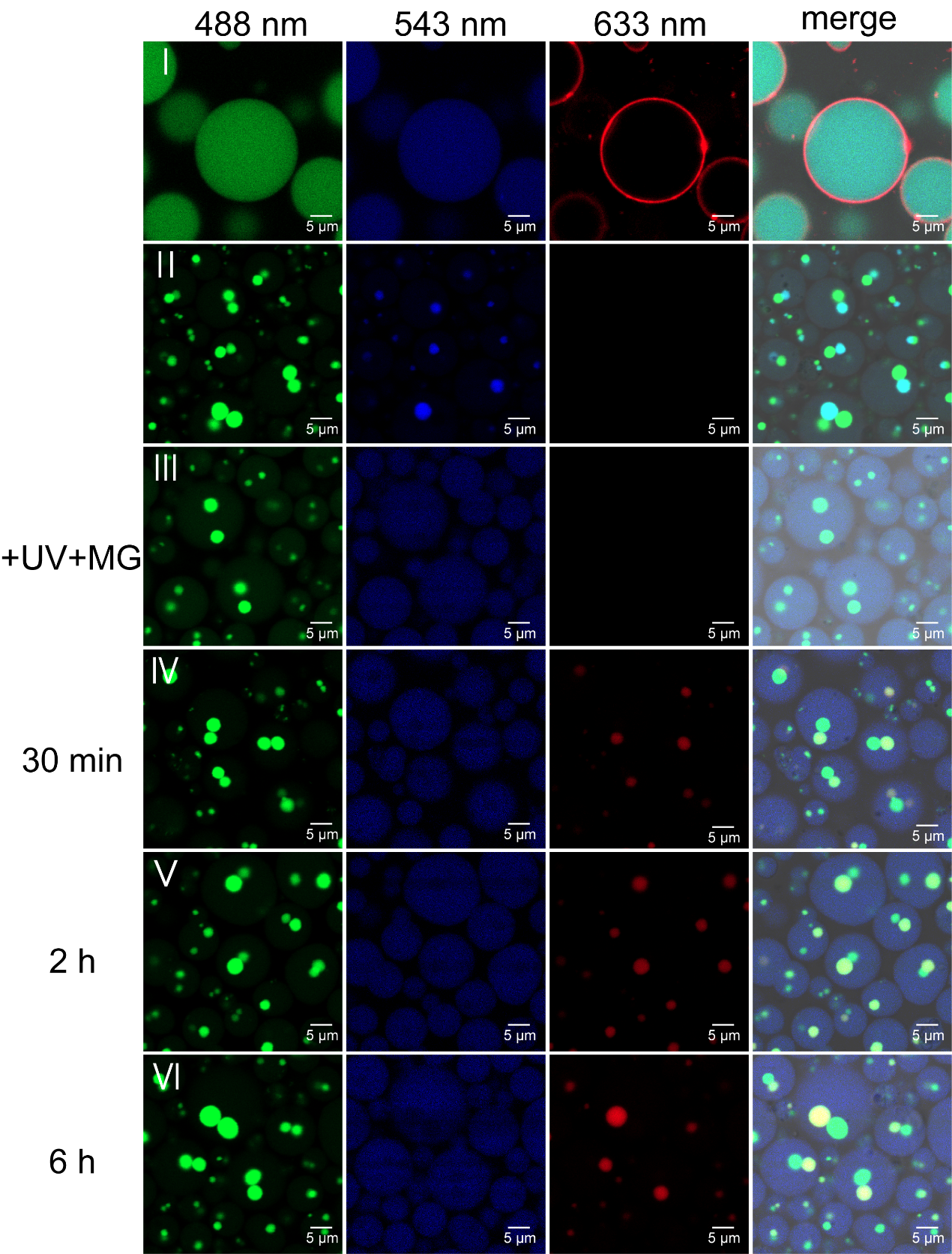


**Figure S26.** Zoom-out confocal fluorescence microscopy images corresponding to: Panel I-the constituents in the pore-locked liposomes, prior to phase separation; Panel II-after unlocking the pores and Mg^2+^-ions induced phase-separated organelles O_7_/O_8_; Panel III-Panel VI-after light-induced activation of organelles O_7_/O_8_ and recording at time-intervals the fluorescence features of the liposome, upon dynamic reconfiguration into the O_7_’/O_8_’ state (Panel III-after 0 min, Panel IV-after 30 min, Panel V-after 2 h, Panel VI-after 6 h,). (d) Time-dependent integrated fluorescence intensities of the red MG-RNA aptamer fluorescence upon dynamic formation of organelle O_7_’. FAM: 488 nm, Green; Cy3: 543 nm, Blue; Cy5 or MG/aptamer: 633 nm, red.

Figure S26 depicts the zoom-out confocal fluorescence microscopy images of light-induced transcription machinery-guided, intercommunication and reconfiguration between the organelles O_7_ and O_8_ in the liposomes of different configurations, c.f. Figure 6(c).

**Probing the stability of the organelle-loaded liposomes in cell medium.**


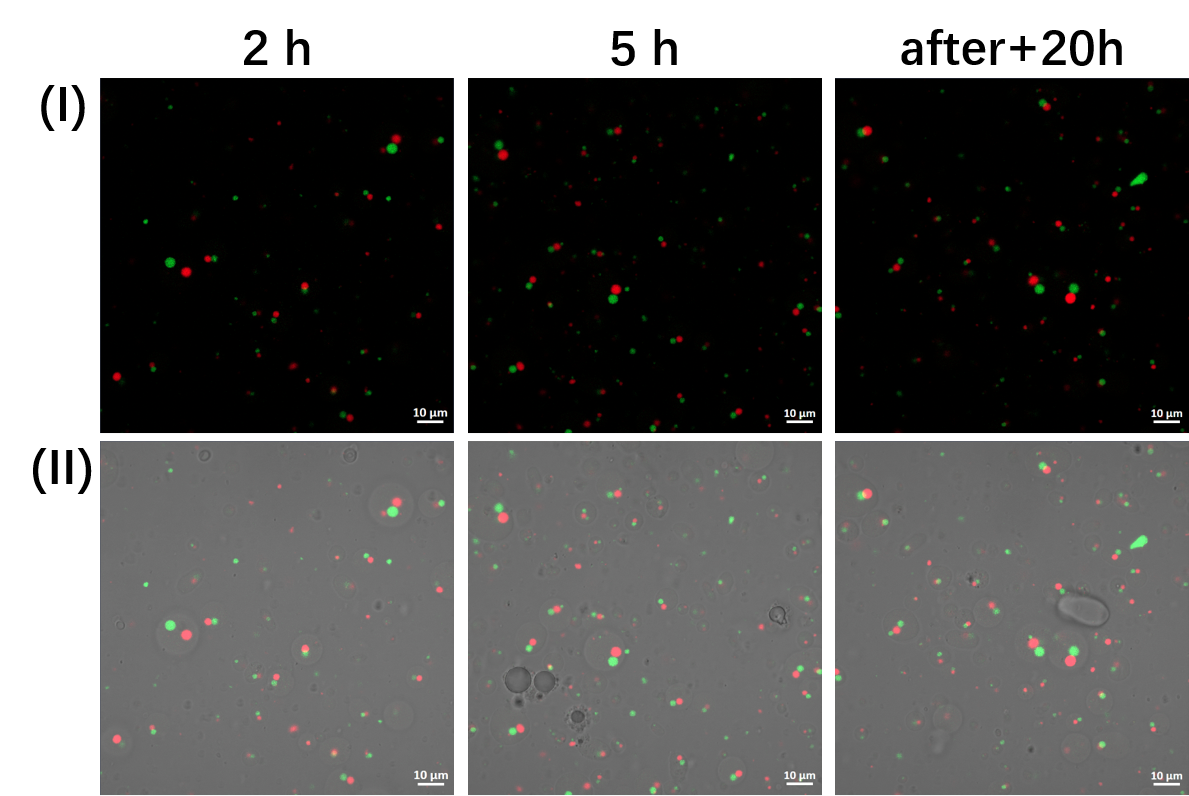


**Figure S27.** Confocal fluorescence microscopy images corresponding to the Organelle O_1_/O_2_ loaded liposomes in the cell culture medium, at different time intervals

The results demonstrate that the O_1_/O_2_-loaded liposome retain the integrated structural feature for at least 24 hours.
